# Mutation Discovery in Regions of Segmental Cancer Genome Amplifications with CoNAn-SNV: A Mixture Model for Next Generation Sequencing of Tumors

**DOI:** 10.1371/journal.pone.0041551

**Published:** 2012-08-16

**Authors:** Anamaria Crisan, Rodrigo Goya, Gavin Ha, Jiarui Ding, Leah M. Prentice, Arusha Oloumi, Janine Senz, Thomas Zeng, Kane Tse, Allen Delaney, Marco A. Marra, David G. Huntsman, Martin Hirst, Sam Aparicio, Sohrab Shah

**Affiliations:** 1 Department of Molecular Oncology, BC Cancer Agency, Vancouver, British Columbia, Canada; 2 Michael Smith Genome Sciences Centre, British Columbia Cancer Agency, Vancouver, British Columbia, Canada; 3 Department of Computer Science, University of British Columbia, Vancouver, British Columbia, Canada; 4 Centre for Translational and Applied Genomics, British Columbia Cancer Agency, Vancouver, British Columbia, Canada; 5 Department of Pathology and Laboratory Medicine, University of British Columbia, Vancouver, British Columbia, Canada; Baylor College of Medicine, United States of America

## Abstract

Next generation sequencing has now enabled a cost-effective enumeration of the full mutational complement of a tumor genome—in particular single nucleotide variants (SNVs). Most current computational and statistical models for analyzing next generation sequencing data, however, do not account for cancer-specific biological properties, including somatic segmental copy number alterations (CNAs)—which require special treatment of the data. Here we present CoNAn-SNV (Copy Number Annotated SNV): a novel algorithm for the inference of single nucleotide variants (SNVs) that overlap copy number alterations. The method is based on modelling the notion that genomic regions of segmental duplication and amplification induce an extended genotype space where a subset of genotypes will exhibit heavily skewed allelic distributions in SNVs (and therefore render them undetectable by methods that assume diploidy). We introduce the concept of modelling allelic counts from sequencing data using a panel of Binomial mixture models where the number of mixtures for a given locus in the genome is informed by a discrete copy number state given as input. We applied CoNAn-SNV to a previously published whole genome shotgun data set obtained from a lobular breast cancer and show that it is able to discover 21 experimentally revalidated somatic non-synonymous mutations in a lobular breast cancer genome that were not detected using copy number insensitive SNV detection algorithms. Importantly, ROC analysis shows that the increased sensitivity of CoNAn-SNV does not result in disproportionate loss of specificity. This was also supported by analysis of a recently published lymphoma genome with a relatively quiescent karyotype, where CoNAn-SNV showed similar results to other callers except in regions of copy number gain where increased sensitivity was conferred. Our results indicate that in genomically unstable tumors, copy number annotation for SNV detection will be critical to fully characterize the mutational landscape of cancer genomes.

## Introduction

**Figure 1:**
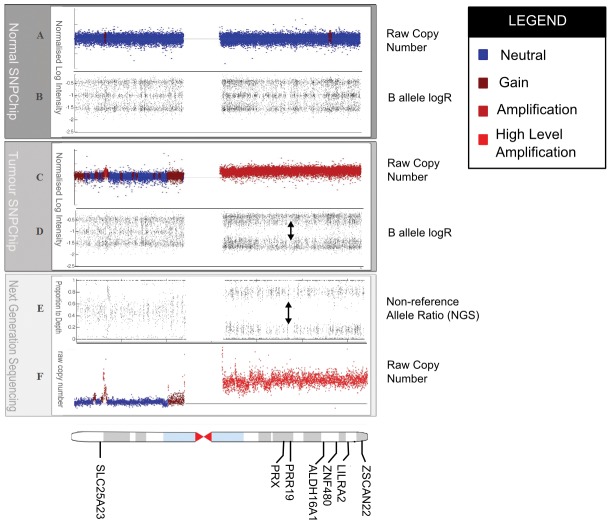
Novel somatic variants detected in allele-specific amplification on chromosome 19q arm. A somatic high-level amplification of the 19q arm is confirmed in NGS as well as Affymetrix SNP6.0 data. Novel somatic variants that were undetectable by samtools variant caller or SNVMix are highlighted on the karyogram. **A**) and **B**) indicate raw log copy number and b allele intensity, respectively, for normal DNA (from the same patient) on Affymetrix SNP 6.0 array. Blue colour indicates diploid (neutral) copy number state; the brighter the colour of red the higher the level of amplification. The three distinct bands in (B) indicate the presence of the alleles harbouring one of the three diploid genotypes: AA,AB and BB. **C**) and **D**) shows metastatic tumor copy number and b allele intensity respectively. The high level amplification on the 19q arm is accompanied by B allele intensities that show an absence of the AB heterozygous (middle) band that was present in the normal. **E**) shows allelic counts from next generation sequencing for the positions represented on the array as a proportion of depth; the allelic ratio is calculated by summing the total number of reads containing a variant at each position divided by the total depth at that position. **F**) shows the raw copy from the NGS data annotated with the amplification information and indicates the same sites of amplification revealed by orthogonal array platform.

Recent advances in massively parallel genome short-read sequencing methods (so-called next generation sequencing (NGS)) have placed the goal of complete delineation of cancer genome landscapes down to single nucleotide resolution within practical reach. New methods for the analysis of short-read sequence data are needed, however, in particular those that are capable of coping with the complex genomic landscapes of tumors. Cancer genomes undergo diverse forms of somatic aberration, including single nucleotide mutations, translocations, gene fusions, deletions, inversions and segmental genome copy number alterations (CNAs). Multiple types of somatic aberration have been reported to occur together: for example, Kadota et al. [1] observed recurrent mutations in *PIK3CA* in breast cancer with allele specific amplifications of the mutant allele in the same tumors and suggested that *PIK3CA* point mutations with concomitant CNA amplification resulted in synergistic oncogenic effects. Similarly, LaFramboise et al. [2] showed allele specific amplification of *EGFR* mutant alleles in a lung cancer cell line; examples of amplification co-occurring with somatic mutations in *MYC* [3], *HRAS* [4], and *MET* [5] have also been observed. The co-occurrence of single nucleotide variants in regions of segmental copy number amplification poses special problems because unknown mixtures of allele abundances could result from the process of segmental amplification and/or subsequent selection, in some cases confounding interpretation. This is because the mixtures of alleles at any one position may be skewed, resulting in a departure from the theoretical frequency (0.5) for heterozygous variants expected in diploid genomes. Figure 1 shows an example from chromosome 19 of a lobular breast carcinoma genome reported in Shah et al. [6] and illustrates a skew in the allelic frequency away from heterozygosity due to an allele-specific copy number amplification on 19q. Both B-allele frequency analysis in the array data and allelic ratio analysis in the NGS data support a mono-allelic amplification on 19q in this genome. We report in this paper that this event harbours 7 co-existing somatic mutations (see Results) in genes (annotated on the karyogram) that are undetectable by analytical methods that assume diploidy. Accurate and sensitive variant calling methods may therefore require conceptual inclusion of co-existing segmental copy number variants (somatic or germline) into the interpretation of measured allele frequencies from NGS data. High density genotyping arrays have allowed for quantification of allele-specific CNAs by incorporating copy number with allelic genotype. Algorithms such as QuantiSNP [7], VanillaICE [8], Birdsuite [9], PennCNV [10] and PICNIC [11] model allele-specific CNAs by extending the genotype state space from the conventional three diploid genotypes: aa (homozygous for major allele), ab (heterozygous) and bb (homozygous for minor allele). For amplified regions the number possible genotypes naturally expand, for example, a triploid chromosome or segmental gain could have the following genotypes: 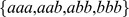. Despite the insights gained through these methods, all are ultimately limited by the resolution and scope of the array design. Most importantly, the discovery of novel somatic point mutations is generally not possible with array platforms. Next generation sequencing overcomes these limitations since whole genome shotgun sequencing (WGSS) can interrogate the entire genome and reveal somatic mutations in loci not covered by arrays. Moreover, the frequency of alleles in a given sample is a digital counting exercise whose dynamic range is not restricted by hybridization and fluorescence intensity saturation and sensitivity constraints.

Several cancer genomes have now been deeply sequenced with NGS and analyzed for CNAs and SNVs independently using bioinformatic approaches followed by targeted validation to confirm somatic alterations. These studies have revealed novel somatic point mutations in acute myeloid leukaemia [12], [13], breast cancer [6], [14], ovarian cancer [15], melanoma [16], lymphoma [17] and lung cancer [18]. Work by Pleasance et al. [16], Chiang et al. [19] and our own work [6] suggest that CNAs can be inferred from sequence data, however none of these studies have used algorithms that explicitly integrate CNAs to inform the inference of SNVs. Here we demonstrate how the incorporation of CNA information in SNV discovery in cancer genome sequence data yields additional novel somatic mutations that were undetectable using conventional SNV prediction algorithms designed for normal diploid genomes.

Studies such as Ding et al. [14] and our own [6] have used ultra deep targeted amplicon sequencing to estimate the frequency of mutations in the population of tumor cells in order to detect sub-dominant or rare clonal cell populations. Here we show that non-diploid allele ratios can also arise from regions of copy number associated disruptions of allelic abundance. We conclude that consideration of copy number results in increased sensitivity to detect both germline and somatic variants in non-diploid regions of cancer genomes.

## Results

### The CoNAn-SNV model

**Figure 2:**
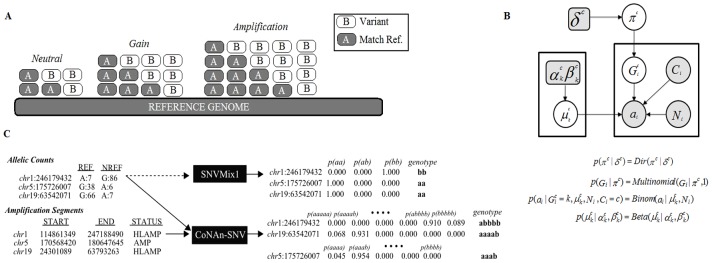
Overview of CoNAn-SNV model, inputs and outputs. **A**) CoNAn-SNV genotype state-space expansion shown schematically. As higher levels of amplification are encountered, a larger genotype state-space is required to accommodate the different events that could arise due to amplifications (examples in Figure S1). **B**) CoNAn-SNV generative probabilistic graphical model. Circles represent random variables, and rounded squares represent fixed constants. Shaded nodes indicate observed data, such as allelic counts, while white nodes indicate quantities that are inferred during training though expectation maximisation. 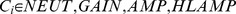 represents the CNA states of a segment (defined by the HMM describe in Shah et al. [6]) that spans position *i*; 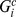 represents the genotype, which varies depending on CNA state; 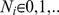 is the number of reads and 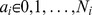 is the number of reference reads; 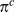 is prior existing over the genotypes and extends to accommodate CNA states; and 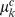 is the genotype-specific Binomial parameter for genotype k in CNA state Ci. C) Example of CoNAn-SNV input and output. CoNAn-SNV takes allelic counts and as well is CNA segment data as input, while SNVMix requires only allelic counts. The same positions and counts are provided to both algorithms, with different results. In some cases CoNAn-SNV will call a variant with an *aaaab* or *aaab* genotype, which would otherwise be missed by SNVMix; also, however, CoNAn-SNV will also genotype a positions with *abbbb* rather than *bb* (as SNVMix [21] would), which allows for better interpretation of events.

To address the problem of allelic states in regions of copy number aberration, we developed a new model, CoNAn-SNV, designed to incorporate knowledge of copy number state at individual positions. Depicted schematically in Figure 2A, and as a generative probabilistic graphical model in Figure 2B, the model uses a hierarchical Bayes [20] conditional independence framework for parameter estimation and inference. CoNAn-SNV relates to the SNVMix1 model described in Goya et al. [21], but with important differences; namely that SNVMix1 does not encode copy number changes commonly found in cancer genomes (such as the 19q amplification shown in Figure 1). To overcome this limitation, CoNAn-SNV inputs a set of allelic counts and a discrete copy number state for each position in the data. An example of the inputs and output is shown in Figure 2C. The objective is to predict which, out of a fixed number of genotypes (informed by the copy number state), would be most likely to have given rise to the observed allelic counts at a given position. The allelic counts are represented as the number of reads 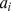 at each position 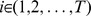 that match the reference, where *T* is the total number of positions in the input. We let 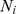 represent the total number of reads aligned to position *i* (or the depth) in the input. We introduce 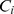 as the copy number state at position *i*, and we assume 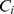 is known at run time. Theoretically, the full space of allele states could be inferred with knowledge of absolute copy number, however methods for determination of absolute copy number from aCGH data remain problematic and in practice it is unlikely that all states could be resolved even with the current sampling depths of NGS (see Discussion). Therefore to a first approximation, we have defined copy number state, 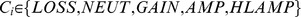, where LOSS corresponds to a deletion, NEUT is copy number neutral, GAIN approximates to low level duplication, AMP approximates to low-intermediate amplification and HLAMP is a high-level copy number amplification. Here we use the HMM-based method described by [6]. They key intuition in the CoNAn-SNV model is that 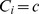 informs the state space of possible genotypes 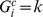 at position *i* as follows:
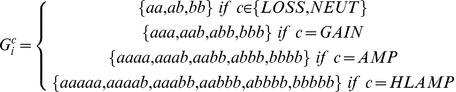
(1)Loss segments are analysed with a neutral state-space because they present challenges that require considerations that are separate from amplifications and in fact may even require a complimentary normal genome. Accounting for copy number gains is especially important when such changes are allele specific, and when the allele that is amplified is the reference allele. For example, consider the case where 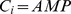, this will induce a genotype state space of 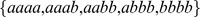. Our model is therefore theoretically capable of detecting variants with allelic distributions skewed away from heterozygosity (i.e. *aaaab* or *abbbb*). We let 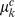 represent the parameter of the Binomial distribution that encodes the expected proportion of reads matching the reference sequence, for a given copy number state 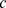 and genotype state 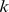. We can therefore express the likelihood for observing the number of reference reads given the depth, the copy number state, the genotype and the model parameters as follows:
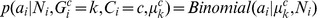
(2)thereby assuming that 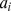 is distributed according to the state-specific Binomial distribution indexed by genotype *and* copy number. We also encode a copy-number specific prior over genotypes 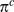, assuming that the genotypes for copy number state c are distributed according to a Multinomial distribution with parameter 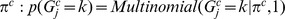 for all 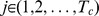, where 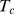 is the total number of positions with copy number state 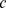. We use Bayes' rule to compute the posterior probability that genotype *k* gave rise to the observed data with the explicit encoding of copy number state:
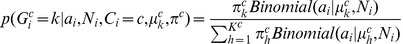
(3)where 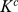 is the number of possible genotypes for copy number state *c* (see Equation (1)). Given 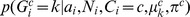, we can then choose to compute:
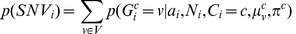
(4)where 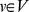 represents any variant genotype state (i.e. any state that is not *aa*, *aaa*, *aaaa*, etc. as the case may be) to represent a single probability that a position encodes a SNV.

#### Hyperpriors and hyperparameters

We assume 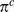 is distributed according to a conjugate Dirichlet distribution with parameters 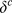. This is a user-defined parameter. In our study we set 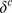 in order to favour non-variant states since most positions in the genome will be homozygous for the reference sequence (i.e. wild-type). We assume 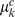 is distributed according to a conjugate Beta distribution with parameters 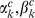. We set 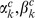 using the biological intuition that homozygous reference positions will be nearly ‘pure’, with decreasing proportion towards homozygous variant positions. All hyperparameter settings are given in Table S1.

#### Model fitting and parameter estimation

Given the free model parameters 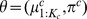, we can showed how to use Equations (3) and (4) to infer for all *i* in the input data. As we showed in [21], it is advantageous to fit the model to the data using expectation maximization (EM) to learn 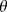. For CoNAn-SNV, we treat the data in each copy number state separately and run EM for each set of data independently (see Methods). We describe it briefly here. Let 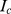 represent the complete set of positions in the input data annotated with copy number state *c*. Iterating over the copy number states 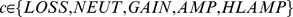, the E-step consists of computing 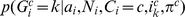 using Equation (3) for each position 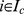, and the current estimates of 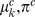. The M-step re-estimates 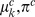 with standard conjugate updating:
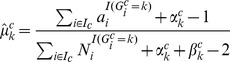
(5)

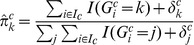
(6)The algorithm continues until the complete data log posterior no longer increases or a maximum number of iterations has been reached.

#### CoNAn-SNV performance on simulated data

We simulated approximately 1000 positions for each copy number state to train the model and then evaluated performance in 100 simulated test sets, which also featured 1000 positions per copy number state. Positions were simulated according to a binomial distribution, where 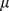 was derived from the hyperparameters described in Table S1, with depth simulated from a Poisson distribution. The distribution of genotypes in each of the simulated copy number states were randomly sampled according to 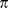 (also calculated from the hyperparameters). The average AUC and 95% confidence intervals, along with the sensitivity at three different false positive rate values (0.01,0.05, and 0.1) were calculated for each CNA-state and are shown in Table S2. CoNAn-SNV and SNVMix had nearly identical performance in the different copy number states, however CoNAn-SNV had improved sensitivity in the highest CN state. For CN state 5, at false positive rate values of 0.01, 0.05 and 0.1, CoNAn had a mean sensitivity of 0.77, 0.84 and 0.88 whereas SNVMix had sensitivity of 0.72, 0.78 and 0.82. These results were not statistically significant, but they establish marginal improvement of CoNAn-SNV over SNVMix without any loss of specificity.

### Experimental validation of the CoNAn-SNV model

**Figure 3:**
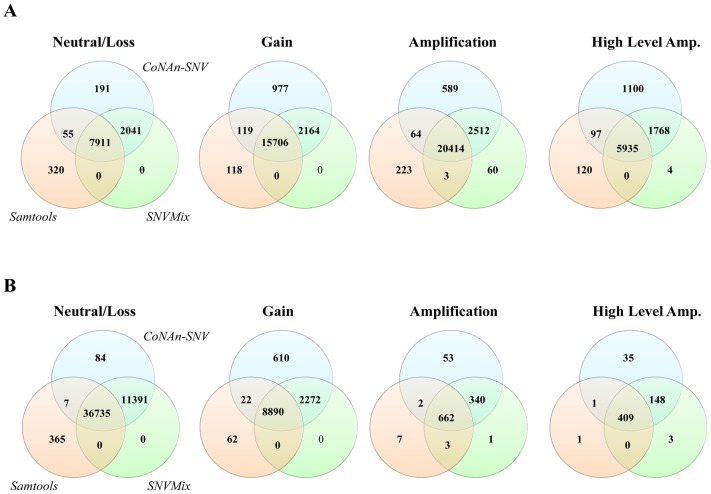
Venn diagram of predictions made by samtools, SNVMix, CoNAn-SNV. Separating by CNA state shows an enrichment of CoNAn-SNV specific predictions in the GAIN, AMP and HLAMP segments of the genome.

To determine the sensitivity and specificity of CoNAn-SNV on real tumour data, we applied the model to the metastatic lobular carcinoma previously published in [6] and subsequently re-sequenced all the novel predictions made by the model to establish its accuracy. The genome was segmented into discrete CNA segments using a hidden Markov model as described in [6] and exhibited a variable CNA landscape. As reported previously, 30.2% of the genome was predicted as loss/neutral, 44.5% was gain, 19.1% amplification and 4.2% high-level amplification (see Table S3). The copy number profile was consistent with the data from that derived from the Affymetrix Snp6 genotyping array (Figure 1) confirming that predicted regions of copy number variations were not induced by the Illumina sequencing platform. Figure 1 shows chromosome 19 and highlights an example of a somatic high level amplification on the 19q arm that also demonstrates a skew in the allelic frequency, away from heterozygosity, due to an allele-specific copy number amplification. Both B-allele frequency analysis in the array data and allelic ratio analysis in the NGS data support a mono-allelic amplification on 19q in this genome. A re-analysis of the genome with CoNAn-SNV made a total of 61,643 SNV calls in exonic regions of the genome (NCBI build 36.1, Ensembl v51 annotations); compared against 58,518 predictions by SNVMix [21] and 51,085 with the samtools mpileup variant caller [22]. Figure 3 shows overlap between CoNAn-SNV, samtools and SNVMix predictions. A total of 49,966 predictions were common to all three methods suggesting reasonable overall agreement. However, 2,857 predictions were CoNAn-specific. In contrast, only 781 positions were specific to samtools and 64 were specific to SNVMix. Figure 3A shows the overlaps between CoNAn-SNV, samtools and SNVMix. Neutral regions harboured 191 CoNAn-specific predictions while Gain, Amplification and High Level Amplifications harboured 977, 589 and 1100 CoNAn-specific predictions respectively. Interestingly, CoNAn-SNV called more SNVs in the neutral states compared with SNVMix despite sharing a common framework. We propose that explicit consideration of CNAs in training procedures allows for better estimation of parameters which would otherwise be influenced by allelic skew in amplified regions (see Methods). SNVs in regions of AMP of HLAMP called by SNVMix and not by CoNAn-SNV had low depths. These low depth sequences in regions of AMP and HLAMP may reflect limits the resolution of the copy number algorithm. At such low depth the binomial likelihoods, for the larger number of allele-specific copy number genotypes, overlap thereby placing more emphasis on the prior to call the final genotype (which biased towards homozygous reference genotype).

**Figure 4:**
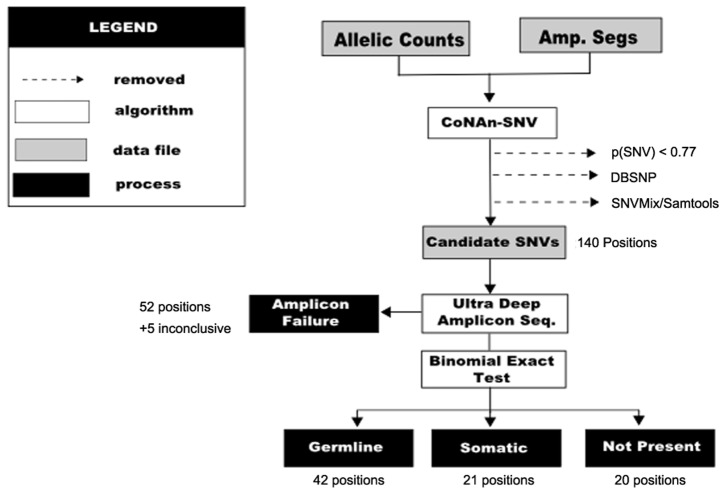
Discovery Flow Diagram.

Figure 3A shows there was a substantial enrichment of CoNAn-specific SNVs in CNA amplification states. From the complete list of 2,857 CoNAn-specific predictions, we filtered out any positions that were present in dbSNP v130 and subsequently identified a set of 140 protein coding, non-synonymous substitution SNVs candidates for validation by targeted, ultra deep amplicon sequencing (shown schematically in Figure 4) in the metastatic and primary (from nine years earlier) tumor genome DNA as well as the normal buffy coat genome DNA from the same patient. A total of 52 SNVs could not be resolved due to PCR amplicon failure during validation, leaving 88 remaining for further analysis. Table 1 shows 21/125 (23.9%) novel, coding, non-synonymous somatic mutations that were validated by deep amplicon sequencing. For all of these somatic variants, their predicted genotypes were highly skewed towards the reference allele and had a most probable genotype of aab, aaab or aaaab (Table 1). These amplicons generated an average of 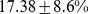 reads representing the mutant allele in the metastatic genome (with a mean depth of coverage of 96,669) whereas the normal genome for the amplicons had an average mutant allele frequency of 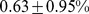 and a mean depth of coverage of 71,963. Note that only one somatic mutation, K187M in ZNF607, a zinc finger protein putatively involved in transcriptional regulation, was also confirmed in the primary tumor. This supports the conclusion from [6] that only few mutations present in the metastatic tumor were present in the primary at diagnosis, and thus were candidate drivers of tumorigenesis. Additionally, we identified 42 (47.7%) germline variants, where the SNV was present in both the normal and metastatic DNA. Finally, 20 (22.7%) positions failed to validate as SNVs and were considered false positive predictions. Five positions (5.68%) were inconclusive because the disparity in depth of coverage between the normal and metastatic tumor validation data was too large to draw conclusions. A full summary of all 140 positions is available in Table S4. The potential functional impact of each of the 21 somatic mutations was assessed using MutationAssessor (http://mutationassessor.org), and is presented in the supplemental material.

**Table 1:** Novel somatic variants identified by CoNAn-SNV.

				WGSS ANALYSIS						NORMAL VALIDATION		PRIMARY VALIDATION		METASTATIC VALIDATION		Transcriptome			
ChromPos	AA Mutation	Gene Name	Impact	Ref Base	Mut Base	Depth	Nref count	p(snv)	Genotype	Depth	Freq. Nref	Depth	Freq. Nref	Depth	Freq. NRef	Ref.	Ref. Count	Nref.	Nref. Count
**1**:31870778	S177L	PEF1	1.11(M)	G	A	17	3	0.8459	aab	11190	0.0441	617	0.0340	27129	0.4731	G	2	N	0
**1**:181813423	Q100H	NCF2	0.975(L)	C	G	85	10	1.0000	aaaab	39290	0.0039	1733	0.0069	60040	0.0977	C	25	N	0
**1**:200099346	R539T	IPO9	2.025(H)	G	C	101	8	0.8015	aaaab	18800	0.0026	274	0.0036	12231	0.0916	G	84	C	11
**2**:100965216	E525T	NPAS2	1.68(M)	A	T	54	7	0.8824	aaab	131465	0.0017	15627	0.0022	187617	0.1796	A	29	T	3
**4**:25524942	S 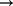 -¿C	AC133961.3	No Uni ID.	C	G	11	2	0.7930	aab	10999	0.0025	443	0.0045	15554	0.2468	N	0	N	0
**5**:175726007	E68K	ARL10	0.55(L)	G	A	51	9	0.9988	aaab	35722	0.0011	5911	0.0008	56243	0.1454	G	1	N	0
**5**:176953851	E152*	TMED9	Truncating	G	T	55	9	0.9962	aaab	83887	0.0110	40283	0.0109	97795	0.2028	G	111	T	9
**6**:44361861	E222K	TCTE1	0.955(L)	C	T	28	5	0.8956	aaab	63261	0.0054	4076	0.0064	70470	0.2327	N	0	N	0
**6**:111800869	N1794K	REV3L	0.345(L)	G	T	36	6	0.9933	aab	91581	0.0016	54683	0.0020	74407	0.2006	G	18	T	3
**6**:157570350	R2115Q	ARID1B	1.845(M)	G	A	52	9	0.9987	aaab	304781	0.0024	118051	0.0022	449145	0.2051	N	0	N	0
**7**:139446250	L561V	JHDM1D	0.615(L)	G	C	41	6	0.9647	aaab	305	0.0000	1	0.0000	137	0.2774	G	91	C	30
**11**:2383399	I109F	TRPM5	−0.08(N)	T	A	20	5	0.9882	aaab	100659	0.0045	33904	0.0104	182328	0.1948	N	0	N	0
**14**:93999360	V359V	SERPINA9	0.28(L)	G	A	28	4	0.7858	aaab	61006	0.0219	8291	0.0226	73354	0.2324	N	0	N	0
**14**:100417938	V982I	RTL1	0805(L)	C	T	33	7	0.9962	aaab	107685	0.0135	6172	0.0146	102285	0.1799	N	0	N	0
**19**:6403457	G313S	SLC25A23	1.83(M)	C	T	15	3	0.9965	aab	46019	0.0048	6579	0.0050	43855	0.2087	C	1	T	2
**19**:42881337	K187M	ZNF607	NA	T	A	77	10	0.9922	aaaab	2722	0.0026	174	0.1667	13589	0.1525	T	15	A	1
**19**:47506592	E24*	PRR19	Truncating	C	T	50	7	0.9674	aaaab	47838	0.0018	2712	0.0026	53450	0.1260	C	5	N	0
**19**:54648470	Q311*	ALDH16A1	Truncating	G	T	52	7	0.9522	aaaab	75066	0.0036	1935	0.0078	91868	0.1159	N	0	N	0
**19**:57509248	E16Q	ZNF480	1.67(M)	G	C	64	11	0.9999	aaaab	16867	0.0033	1133	0.0071	52154	0.0862	G	12	C	1
**19**:59779115	V328M	LILRA2	1.91(M)	G	A	53	8	0.9922	aaaab	145106	0.0029	60245	0.0028	264119	0.1177	G	6	N	0
**19**:63542071	G348E	ZSCAN22	2.99(H)	G	A	71	8	0.9201	aaaab	279784	0.0023	64866	0.0021	218744	0.0996	G	1	N	0

Somatic variants that were uniquely predicted by CoNAn-SNV and were successfully validated by targeted ultradeep amplicon sequencing.Impact refers to functional impact as determined by MutationAssessor.

* Refers to a stop codon.

Sub-heterozygous allele abundance could result from sub-dominant populations of cells or unequal allele amplification in regions of copy number aberration. For example, preferential copy number associated amplification of a wildtype allele would result in less than heterozygous ratios of a somatic mutant allele. Notably, the mean abundance of the novel somatic SNVs from the validation experiments above, was 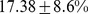 with four mutations (affecting genes *NCF2*, *IPO9*, *ZNF480* and *ZSCAN22*) exhibiting a proportion of less than 10%. Without consideration of the copy number status, the probability of a non-reference event would be down-weighted, leading to loss of sensitivity. Furthermore, germline allelic ratios could help confirm whether the copy number segment involved is predominantly mono-allelic. We examined the allelic ratios for all informative positions in the CNA segments analysed. We found seventeen of the 42 validated germline variants also exhibited substantial allelic skew, as highlighted in Table 2 (see Methods). Notably, germline variants at positions chr19: 40691038, chr19:42074256, chr19:50869860 and chr19:59415177 within the high level amplicon on chr19 had allelic distributions in the tumour that were skewed significantly away from their normal distribution (Chi Sq test, 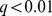). These germline SNPs are proximal to the somatic mutations K187M in *ZNF607*, E24* in *PRR19*, Q311* in *ALDH16A1*, E16Q in *ZNF480*, V328M in *LILRA2*, and G348E in *ZSCAN22*. The most parsimonious explanation for these findings is that the somatic mutations were a later event, however it is not known if they occur on one of the amplified chromosomes or the residual unamplified sister chromosome. A different validation procedure would be required to make this inference. This is supported by an additional 424 SNVs within the 19q high level amplicon (chr19: 24301089–63793263 see Table S5) that were predicted to be either aaaab or abbbb by CoNAn-SNV but were not sent for revalidation. The enrichment of skewed *germline* alleles in regions of significant copy number change renders the possible explanation of allelic skewing of somatic variants in the same regions due to tumour-normal admixture extremely unlikely. Finally, the OncoSNP http://groups.google.co.uk/group/quantisnp/web/downloads-oncosnp algorithm predicted an unbalanced amplification spanning chr19:32439833–63789666 (Figure S1) in the corresponding Affymetrix SNP 6.0 data. This segment was predicted by OncoSNP to contain 638 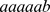 variants, and 591 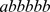 variants, supporting the conclusion of an allele-specific amplification in 19q. Interestingly, the allelic frequency of K187M in *ZNF607*, the only somatic variant found in the primary tumor (16.67%) was consistent in the metastatic tumor (15.25%), suggesting that the other 19q mutations occurred later in the tumor evolution.

**Table 2:** Effect of copy number amplifications on germline alleles.

			Normal		Metastatic		Transcriptome				
ChromPos	AA mutation	Gene	Depth	Freq. Nref	Depth	Freq. Nref	Ref.	Ref. Count	Nref.	Nref. Count	Chi sq. q-value
**1**:144932587	F218C	AL139152.7	17928	0.3169	18017	0.2164	T	55	G	3	1.27E-102
**1**:149999951	I213V	MRPL9	5387	0.2046	8770	0.0409	T	154	C	28	4.29E-211
**1**:150543396	R3530S	FLG	61790	0.6191	78410	0.3981	N	0	N	0	0
**8**:146033676	A76V	ZNF7	92012	0.4499	147007	0.2683	C	2	N	0	0
**9**:33375641	C 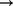 F	AQP7	24722	0.2781	22104	0.1985	N	0	N	0	1.12E-89
**10**:29823914	M1259T	SVIL	128591	0.3867	110884	0.4808	A	6	N	0	0
**11**:390124	N477K	PKP3	37172	0.4601	57560	0.2907	C	11	N	0	0
**11**:17499485	R357Q	USH1C	101208	0.5595	58749	0.1548	N	0	N	0	0
**11**:65860057	A79T	RIN1	75400	0.4044	97848	0.1738	N	0	N	0	0
**11**:116569101	R710C	SIDT2	260320	0.5342	237372	0.1390	C	51	T	19	0
**11**:124827464	E358Q	FEZ1	249388	0.5259	171924	0.1372	C	0	G	2	0
**12**:122455439	R279P	STED8	208542	0.3071	175257	0.4182	G	17	N	0	0
**17**:36549887	S 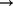 P	KRTAP4-15	1774	0.3207	4409	0.1851	N	0	N	0	1.51E-30
**19**:40691038	R 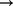 Q	DMKN	209119	0.5478	247223	0.1696	C	5	T	2	0
**19**:42074256	H426R	ZNF829	6402	0.4531	10867	0.1214	T	1	C	1	0
**19**:50869860	R190Q	GIPR	70793	0.4878	90262	0.1843	G	26	A	5	0
**19**:59415177	R 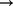 K	LILRB3	34753	0.1592	46500	0.0642	N	0	N	0	0

These variants exhibit an amplification of the reference allele and show allelic skew, and as a result suggest an unbalanced allelic amplification over the tumor evolution.Impact refers to functional impact as determined by MutationAssessor.

#### CoNAn-SNV retrieves more true positives without compromising overall accuracy

We assessed performance by evaluating the area under receiver operator characteristic curve (AUC) for CoNAn-SNV and SNVMix. The positions used as the ground truth were obtained from an Affymetrix SNP 6.0 positions genotyped using CRLMM [23] and additionally with OncoSNP (see Methods). Although high confidence CRLMM calls had served as sufficient benchmark for SNVMix in [21], it is important to note that CRLMM assumes diploidy and its calls will therefore be enriched for heterozygous positions that approach expected allelic distributions for diploid genomes. OncoSNP, conversely, extends its state-space to accommodate genotypes induced by CNA events and can therefore capture allele-specific amplifications. As previously noted, OncoSNP calls were concordant with the NGS data and supported that notion that chromosome 1 and 19 have allele-specific amplifications (Table S6 and Figure S1).

**Figure 5:**
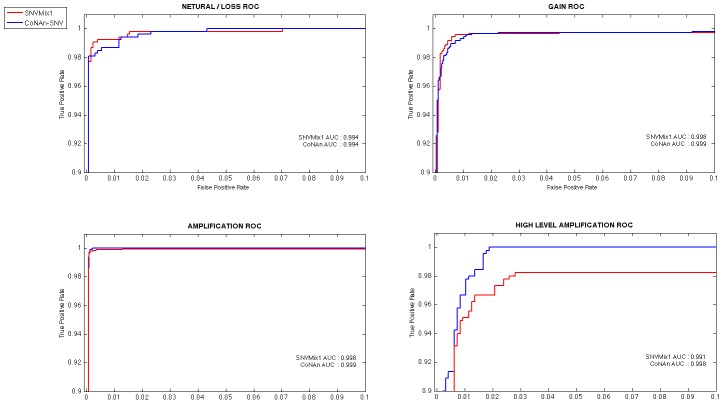
Receiver operator characteristic curve for CoNAn-SNV and SNVMix broken down by amplification status.

The ROC results for OncoSNP suggest that CoNAn-SNV and SNVMix perform similarly, except in regions of high-level amplifications (see Figure 5). The AUCs for SNVs in regions of GAIN was 0.998 for SNVMix and 0.999 for CoNAn-SNV. For amplification and high-level amplification, the AUCs were (0.998, 0.999) and (0.991, 0.998) respectively. Examination of the breakdown of the calls (Table S7) we determine that CoNAn-SNV calls more true positives overall, compared with SNVMi1, which was also observed in the simulation data set, but is also subject to calling more false positives. The proximity of the AUC measurements suggests that the false positives introduced by CoNAn-SNV do not outweigh the additional true positives retrieved. The ROC for HLAMP is very different from the others, due to SNPs harboured in the allele-specific CNA regions of chromosome 1 and 19 that could not be detected by SNVMix.

CRLMM results are a benchmark for variants that are easy to detect by SNVMix. Area under ROC curve calculations indicated that CoNAn-SNV performs similarly to SNVMix for these positions (Figure S2). The AUC for SNVs in regions of GAIN was 0.979 for SNVMix and 0.975 for CoNAn-SNV. For amplification and high-level amplification, the AUCs were (0.991, 0.990) and (0.911, 0.928) respectively. This suggests that the increased sensitivity gained by CoNAn-SNV does not compromise its overall accuracy compared to SNVMix, which was also demonstrated using OncoSNP to assess performance.

#### CoNAn-SNV performance on a quiescent tumor

The genomic landscape of a tumor varies across different cancer types. CoNAn-SNV is applicable to tumours with quiescent genome architectures as well as those with more disrupted karyotypes; to demonstrate this we evaluated CoNAn-SNV's performance in a lymphoma tumor originally published in Morin et al [24] where 71.9% of the genome was predicted as loss/neutral, 22.1% was gain, 4.30% amplification and 1.67% high-level amplification (see methods). We used CoNAn-SNV, SNVMix as well as the samtools to profile the mutational landscape of the lymphoma tumor genome; each method found 62,162, 61,352 and 47,164 variants respectively(Figure 3B). For this tumour, an approximate 30× coverage WGSS dataset of the matched normal DNA was available, thereby permitting the ascertainment of somatic mutations directly from the data itself. A total of 782 variants were unique to CoNAn-SNV, otherwise there was high agreement between all three methods (Figure S4). We used the mutationSeq software to determine the presence of somatic variants (see Methods). This yielded 392, 365 and 228 somatic mutations for CoNAn-SNV, SNVMix and samtools (Table S8). Of the 228 somatic predictions from samtools, 221 were also found by CoNAn-SNV; and all 365 somatic predictions from SNVMix were found by CoNAn-SNV (Figure S4). The presence of unique somatic variants to CoNAn were nearly exclusively in regions of copy number GAIN (19/22). CoNAn-SNV produced nearly identical results to the diploid methods in diploid/loss regions of the genome,which suggests strongly that modelling copy number confers a sensitivity advantage without loss of specificity, even in relatively diploid karyotypes and that the CoNAn-SNV model should generalise well to tumours with normal karyotypes.

## Discussion

In this study we showed that the explicit integration of CNA information with SNV discovery is an essential step towards the goal of comprehensive mutational profiling by next generation sequencing of cancer genomes. Unbalanced segmental copy number alterations are very frequent in tumor genomes and the presence of an unbalanced amplification or deletion of DNA would result in altered allelic ratios in randomly sampled sequence. Without incorporating this copy number information, probabilistic models of SNV detection cannot adjust their sensitivity accordingly. CoNAn-SNV incorporates copy number information into a Bayesian mixture model framework, using a reduced copy number space with 6 states. The number of possible allelic states naturally expands with increasing copy number, however at the same time, the number of reads required to reliably distinguish all states, will also increase. At high copy number states, distinction between higher order states differing by one allele would be highly impractical. A pragmatic approach is therefore to reduce copy number to 6 states, in our case inferred by a previously published HMM method [6]. To test the sensitivity and specificity of CoNAn-SNV, we first analysed, in silico, the behaviour of the model in comparison with non-CNA aware SNV callers; using the genome of a metastatic lobular breast cancer as ground truth, where many somatic and germline variants have been validated by independent methods. Using the CoNAn-SNV predictions, we validated 21 novel somatic non-synonymous coding mutations predicted using CoNAn-SNV that were not identified in the original analysis of this genome [6]. All of these variants had allelic skew resulting from copy number amplifications of the reference allele, thus their predictions in this analysis can be directly attributed to the extension of the model to consider CNAs in the inference of SNVs. Samtools and SNVMix are capable of identifying allelic skew towards the non-reference allele although would likely characterise such an event with the bb genotype. This may be considered a loss of information, while CoNAn-SNV may classify similar events as 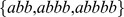 which may provide a more informative description of the genomic landscape at that location and flag certain events as potentially interesting for validation (Table S5). Moreover skewed allelism in simpler models might be misconstrued as loss of heterozygosity. CoNAn-SNV rather allows the investigator to infer skewed heterozygosity caused by allele specific CNAs. Overall, CoNAn-SNV is capable of calling more variants in highly amplified CNAs compared with SNVMix and samtools. Performance metrics indicate that the false positives introduced by CoNAn-SNV do not outweigh the true positives gained. Upon validation of 140 high confidence CoNAn-SNV calls, we resolved that approximately 75.9% of predicated variants (excluding inconclusive and PCR failed results) successfully validated. Of those, there were more predicted variants that validated from so called “high level” CNAs than any others (Table S4). It is possible that this arises from difficulty in establishing the boundaries of the CNA segments which may be too broadly defined; some small lengths of lower level amplifications may exist within other CNAs and the extended state-space is applied where it is not needed and detects noise. A high level CNA has a large enough difference from the background, especially when surrounded by neutral regions, that it may be easier to establish the segmentation boundaries. Although there are still variants that fail to be present, the success of high level amplification predictions and support from surrounding germline variants suggests that CoNAn-SNV framework accurately represents genotypes existing within these regions. The capacity to accurately call a variant is also largely dependent upon the ability of aligners to accurately map a read. Often true variants existing in the data may cause ambiguous alignments which renders reads unusable or incorrectly placed. As aligners continue to progress, we expect the false positive rate and true positives rates of CoNAn-SNV will improve and return more accurate results. Since our software is samtools [22] compliant, the emergence of new, improved aligners that use the samtools community standards will not require any modification to our framework.

Lastly, we applied CoNAn-SNV to a relatively quiescent lymphoma tumor for which both tumour and normal data was available, using mutationSeq as a post-procession tool to predict somatic variants (see Methods). We found that CoNAn-SNV found only an additional 782 variants in the tumor, 22 of which were predicted somatic variants primarily in gain copy number states. In total the CoNAn-SNV had relatively high agreement with SNVMix and samtools diploid variant calling methods. Thus, CoNAn-SNV is applicable to tumor landscapes that are have quiescent or disrupted genomic landscapes.

### Limitations and Future Work

A well known problem with the Binomial probability density function parameterized by 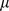 is that it exhibits very narrow peaks with increasing numbers of observations. As such, small deviations from the expected values in regions with substantial depth can produce extremely low likelihoods and uninformative likelihoods for all genotypes. In such cases, the prior probability on genotypes (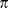) can dominate the calculation of the posterior and over-influence the overall SNV call. The prior probabilities are distributed such that the majority of the probability mass is skewed towards the homozygous reference genotypes. As a result, some true SNVs may not be correctly classified. However, the natural extension of the model to use a Beta-Binomial (overdispersed) likelihood to mitigate against this effect has thus far proven to be no more accurate, and therefore further extensions may be needed. Moreover, the CoNAn-SNV model is restricted to the possible state space of genotypes provided in the input data. Joint and simultaneous inference of copy number and genotype is a theoretically more attractive approach, since genotype could influence the estimation of copy number and vice versa. This would likely improve accuracy should incorrect copy number assignments be used as input into the CoNAn-SNV model. Joint inference however, is substantially more complex and is beyond the scope of this contribution.

### Implications for inference of mutational heterogeneity, tumor evolution and LOH

Our results show on a genome-wide basis how somatic point mutations can overlap with somatic CNAs in a manner that affects their detection and interpretation. Sub heterozygous somatic SNV allele ratios can arise from sub-dominant populations of cells or from masking of the somatic SNV by amplification of the wildtype allele. To resolve this situation, comparisons of tumor genome evolution are required, as shown by us and others [6], [16]. In the latter cases, sub dominant clonal evolution could be inferred because subdominant alleles became prevalent in diploid regions of the genomes or where copy number was not altered during progression. Without the possibility of comparison over time and evolution, skewed allelism in regions of CNA must be cautiously interpreted. Our validation data also showed germline events in CNAs that exhibited allelic skew, as would be expected of an allele specific copy number aberration. Without appropriate consideration of amplification status, these events may have been misconstrued as loss of heterozygosity when in fact the data show that the imbalance results from the amplification of the reference allele rather than hemizygous deletion or copy-neutral LOH events.

### Conclusions

The primary objective of this study was to explore how the consideration of CNA annotation in SNV discovery impacts the analysis and interpretation of NGS data from genomically unstable tumor genomes. We show that explicit integration of copy number information into algorithms of SNV detection not only increases sensitivity, but allows the significance of somatic mutation frequency in diploid and non-diploid regions to be more appropriately interpreted. The discovery of 21 new somatic mutations in the lobular breast cancer reveals how incorporation of CNAs into SNV analysis is essential to approaching comprehensive characterization of the somatic mutational landscape tumours by next generation sequencing technology.

## Methods

Short read sequences that were obtained from the Illumina Genome Analyzer 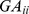 were aligned and analysed using the full analytical pipeline described in Figure S3. All raw data for this study are available through material transfer agreement from the European Genome-Phenome archive (http://www.ebi.ac.uk/ega) under accession number: EGAS00000000054. Lobular breast carcinoma WGSS and WTSS sequence reads were aligned using BWA under default settings. Lymphoma data was aligned by BWA as described in Morin *et al.* [24]. Copy number for the lymphoma genome was determined by HMMCopy (Lai and Shah in preparation), accounting for GC-bias and mappability-bias as described at http://compbio.bccrc.ca/software/hmmcopy.

### Single Nucleotide variants discovery and Validation

The model parameters for CoNAn-SNV were estimated by expectation maximization using 14,649 positions with high confidence SNP calls established as a ground truth standard in [21]. We fit a separate model for each of loss/neutral, gain, amplification and high level amplification sets of positions using expectation maximization in a maximum a posteriori (MAP) framework with hyperparameter settings shown in Table S1. Given the model parameters, we then applied CoNAn-SNV on the full set of WGSS lobular breast carcinoma data. To compute the probability of the presence of a SNV, we summed the posterior probabilities of the variant-containing genotypes (see Equation (4)). We then filtered out any positions where p(SNV) did not exceed the false positive rate threshold determined in [6] of p(SNV)
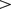
0.77. We use this threshold for accurate comparison against early SNV calls reported in [6]. Remaining positions were filtered against samtools and SNVMix calls as well as dbSNP positions, leaving only CoNAn-SNV specific predictions for further analysis. The final filtration step required that the candidate validation positions to be coding and non-synonymous. A total of 140 positions were submitted for validation by targeted ultra deep amplicon sequencing on the Illumina 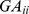 sequencer in the metastatic and primary tumor DNA as well as the normal buffycoat DNA. Details of sample preparation, primer design, library construction and sequencing for validation of the 140 positions are given in Methods S1. A list of the primers is available in Table S9.

All validation sequence reads were aligned using Maq [25] to a custom reference created from the primer coordinates used to generate the amplicons; the reference is available as Supplemental Data in Fasta format. A one-tailed Binomial exact test using the R statistical package was used to evaluate target positions for presence of the SNV against a null distribution designed to capture the background error rate. Allelic counts for the five positions immediately flanking the both sides of the target position on both sides were used to establish the null distribution. Positions that had a Benjamini-Hochberg corrected p-value 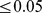 were considered to be present. This procedure was applied to the normal, primary and metastatic data. Positions were considered somatic mutations if they were not present in the normal data, but existed in the tumor data; and germline SNPs if present in the normal and metastatic data. Positions that had a large discrepancy between the metastatic and normal depth, despite Binomial exact test results, were considered inconclusive and were not considered. Some of the germline variants were selected as indicators of allelic skew using a chi-squared test compared the allelic counts of metastatic tumor against the normal. Positions were considered skewed if the Benjamini-Hochberg corrected p-values were 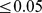 with the additional requirement that the frequency of the non-reference allele between the normal and the metastatic had a disparity of at least 10%.

### Performance Evaluation with OncoSNP and CRLMM

Performance evaluation was completed using an orthogonal Affymetrix SNPChip 6.0 array of the lobular carcinoma. First, we used a well-characterized set of 14649 CRLMM calls as described in [21]. In addition, we analysed the SNP array using OncoSNP in order to benchmark CoNAn-SNV against an analysis capable of detecting allele-specific CNAs (albeit limited to arrays). OncoSNP provided no results for 338,755 positions and these were excluded from analysis. We moved forward with 530,567 OncoSNP calls that were further filtered prior to being used in performance analysis. Overall, there was also a large concordance between CRLMM and OncoSNP genotype calls (498,984 SNP positions) where 15,757 positions were confirmed to be a SNP by both algorithms. A total of 11,369 genotype calls were unique to OncoSNP and mainly represent allele-specific amplifications where the reference allele was amplified; 4,457 were unique to CRLMM likely due to OncoSNP calibration (see below). Since array data reports major-minor allele genotypes and our sequence analysis represent alleles with respect to the reference genome, all array genotypes were adjusted to be compatible with the sequence genotypes. To qualify for further analysis, all positions were required to have a minimum depth of 2, with a minimum mapping and base quality of 10 and 20 respectively. Finally, some positions called a variant by OncoSNP, however the NGS data at the corresponding genomic coordinate lacked evidence of any variant reads. These positions either represented a missed call from OncoSNP or an under-sampling of the allele in the sequence data and thus these positions are removed from analysis so as not to artificially bias the false negative rate. Ultimately, 12,588 positions passed all criteria of which 4,235 were SNVs and 8,353 were not.

### Application to Lymphoma

Tumour and matched normal lymphoma data were cases A03290 and A03291, respectively, selected from [24]. The lymphoma data was subject to the same sequencing and down-stream filtering as the lobular carcinoma data. In place of validating the somatic mutations in the wetlab, we used the mutationSeq software [26] to predict the presence of somatic variants. MutationSeq is a feature based classifier used to detect somatic SNVs from tumour-normal paired data and is robust to germline variants as well as strand bias, mapping quality, base quality, homopolymer run and tail-distance to end of the read induced artifacts [26].

### Implementation and availability

Software for CoNAn-SNV is freely available at http://compbio.bccrc.ca and is implemented in the C programming language. We have compiled and tested the software in the Linux and Mac OSX operating systems. The script for the simulation is also available on the website and was implemented in R.

## Supporting Information

Methods S1 **Validation of SNVs in lobular breast cancer.**
(DOC)

Figure S1 **Copy number annotations for all chromosome made by OncoSNP.**
(PDF)

Figure S2 **ROC for performance evaluation using CRLMM broken down by CNA state.**
(TIF)

Figure S3 **Full variant discovery pipeline.**
(TIF)

Figure S4 **MutationSeq somatic variant results for lymphoma.** Predicted variants with a probability of 0.5 or greater for being a somatic variants (probability assigned by MutationSeq) are shown in a lymphoma tumor for CoNAn-SNV, SNVMix, and the samtools variants caller. There is a high degree of concordance between the three methods, however CoNAn-SNV finds the most unique variants, especially in Gain states.
(TIF)

Table S1 **CoNAn-SNV model parameters.**
(XLS)

Table S2 **SNVMix and CoNAn-SNV simulation comparisons.** AUCs, with 95% confidence intervals (CIs), are calculated for each copy number state over 100 simulation runs. The sensitivity (ad 95% CIs) of SNVMix and CoNAn-SNV is also reported at the following false positives rates: 0.01, 0.05 and 0.1. SNVMix and CoNAn-SNV have a similar sensitivity in NORM and GAIN CNA states, in AMP and HLAMP CoNAn has a much higher sensitivity when compared to SNVMix.
(XLS)

Table S3 **CNA segment input to CoNAn-SNV.** CoNAn-SNV takes as input CNA segments in addition to allelic counts. A line of input indicates a chromosome number, segment start and end site, and lastly a numerical encoding of the CNA state. The numbers are: 2(NEUT/LOSS); 3 (GAIN); 4(AMP); and 5(HLAMP). The model can receive input from any segmentation algorithm so long as it is provided in the same format as this table. Additionally, CoNAn-SNV is not constrained to the state-space used in this paper, and is flexible to other levels of amplification so long as they can be encoded numerically. There are, however, important considerations that should be made if choosing to extend the state-space beyond what has been described in this manuscript. Further instruction for using the model is available on the download page.
(XLS)

Table S4 **Summary of the 140 positions submitted for validation.**
(XLS)

Table S5 **Summary of the 140 positions submitted for validation.**
(XLS)

Table S6 **Genomic Positions with skewed allelic genotypes.** This table indicates positions in the entire genome that harbour the extreme allelic skews such as aaaab and abbbb.
(XLS)

Table S7 **OncoSNP CNA segment predictions.**
(XLS)

Table S8 **Somatic Variants verified by MutationSeq for CoNAn-SNV, SNVMix and samtools.**
(XLS)

Table S9 **Primer specifications for the 140 candidate validation positions.**
(XLS)

## References

[1] Kadota M, Sato M, Duncan B, Ooshima A, Yang HH, et al (2009) Identification of novel gene amplifications in breast cancer and coexistence of gene amplification with an activating mutation of PIK3CA. Cancer Research 69: 7357–7365. 19706770 10.1158/0008-5472.CAN-09-0064 PMC2745517

[2] LaFramboise T, Weir BA, Zhao X, Beroukhim R, Li C, et al (2005) Allele-Specific Amplification in Cancer Revealed by SNP Array Analysis. PLoS Computational Biology 1: 11. 10.1371/journal.pcbi.0010065 PMC1289392 16322765

[3] Herrick J, Conti C, Teissier S, Thierry F, Couturier J, et al (2005) Genomic organization of amplified MYC genes suggests distinct mechanisms of amplification in tumorigenesis. Cancer Research 65: 1174–1179. 15735000 10.1158/0008-5472.CAN-04-2802

[4] Bianchi AB, Aldaz CM, Conti CJ (1990) Nonrandom duplication of the chromosome bearing a mutated Ha-ras-1 allele in mouse skin tumors. Proceedings of the National Academy of Sciences of the United States of America 87: 6902–6906. 1697691 10.1073/pnas.87.17.6902 PMC54646

[5] Zhuang Z, Park WS, Pack S, Schmidt L, Vortmeyer AO, et al (1998) Trisomy 7-harbouring nonrandom duplication of the mutant MET allele in hereditary papillary renal carcinomas. Nature Genetics 20: 66–69. 9731534 10.1038/1727

[6] Shah SP, Morin RD, Khattra J, Prentice L, Pugh T, et al (2009) Mutational evolution in a lobular breast tumour profiled at single nucleotide resolution. Nature 461: 809–13. 19812674 10.1038/nature08489

[7] Colella S, Yau C, Taylor JM, Mirza G, Butler H, et al (2007) QuantiSNP: an Objective Bayes Hidden-Markov Model to detect and accurately map copy number variation using SNP genotyping data. Nucleic Acids Research 35: 2013–25. 17341461 10.1093/nar/gkm076 PMC1874617

[8] Scharpf RB, Parmigiani G, Pevsner J, Ruczinski I (2008) Hidden Markov models for the assessment of chromosomal alterations using high-throughput SNP arrays. The annals of applied statistics 2: 687–713. 19609370 10.1214/07-AOAS155 PMC2710854

[9] Korn JM, Kuruvilla FG, McCarroll SA, Wysoker A, Nemesh J, et al (2008) Integrated genotype calling and association analysis of SNPs, common copy number polymorphisms and rare CNVs. Nature Genetics 40: 1253–1260. 18776909 10.1038/ng.237 PMC2756534

[10] Wang K, Li M, Hadley D, Liu R, Glessner J, et al (2007) PennCNV: An integrated hidden Markov model designed for high-resolution copy number variation detection in whole-genome SNP genotyping data. Genome Research 17: 1665–1674. 17921354 10.1101/gr.6861907 PMC2045149

[11] Greenman CD, Bignell G, Butler A, Edkins S, Hinton J, et al (2010) PICNIC: an algorithm to predict absolute allelic copy number variation with microarray cancer data. Biostatistics Oxford England 11: 164–175. 10.1093/biostatistics/kxp045 PMC2800165 19837654

[12] Ley TJ, Mardis ER, Ding L, Fulton B, McLellan MD, et al (2008) DNA sequencing of a cytogenetically normal acute myeloid leukaemia genome. Nature 456: 66–72. 18987736 10.1038/nature07485 PMC2603574

[13] Mardis ER, Ding L, Dooling DJ, Larson DE, McLellan MD, et al (2009) Recurring mutations found by sequencing an acute myeloid leukemia genome. The New England Journal of Medicine 361: 1058–1066. 19657110 10.1056/NEJMoa0903840 PMC3201812

[14] Ding L, Ellis MJ, Li S, Larson DE, Chen K, et al (2010) Genome remodelling in a basal-like breast cancer metastasis and xenograft. Nature 464: 999–1005. 20393555 10.1038/nature08989 PMC2872544

[15] Shah SP, Köbel M, Senz J, Morin RD, Clarke BA, et al (2009) Mutation of FOXL2 in granulosa-cell tumors of the ovary. The New England Journal of Medicine 360: 2719–2729. 19516027 10.1056/NEJMoa0902542

[16] Pleasance ED, Stephens PJ, O'Meara S, McBride DJ, Meynert A, et al (2010) A small-cell lung cancer genome with complex signatures of tobacco exposure. Nature 463: 184–190. 20016488 10.1038/nature08629 PMC2880489

[17] Morin RD, Johnson NA, Severson TM, Mungall AJ, An J, et al (2010) Somatic mutations altering EZH2 (Tyr641) in follicular and diffuse large B-cell lymphomas of germinal-center origin. Nature Genetics 42: 181–5. 20081860 10.1038/ng.518 PMC2850970

[18] Pleasance ED, Cheetham RK, Stephens PJ, McBride DJ, Humphray SJ, et al (2010) A comprehensive catalogue of somatic mutations from a human cancer genome. Nature 463: 191–6. 20016485 10.1038/nature08658 PMC3145108

[19] Chiang DY, Getz G, Jaffe DB, O'Kelly MJT, Zhao X, et al (2009) High-resolution mapping of copy-number alterations with massively parallel sequencing. Nature Methods 6: 99–103. 19043412 10.1038/nmeth.1276 PMC2630795

[20] Gelman A, Carlin JB, Stern HS, Rubin DB (2003) Bayesian Data Analysis, Second Edition. Chapman & Hall/CRC, 668 p. pp. URL http://www.amazon.com/dp/158488388X.

[21] Goya R, Sun MGF, Morin RD, Leung G, Ha G, et al (2010) SNVMix: predicting single nucleotide variants from next-generation sequencing of tumors. Bioinformatics 26: 730–736. 20130035 10.1093/bioinformatics/btq040 PMC2832826

[22] Li H, Handsaker B, Wysoker A, Fennell T, Ruan J, et al (2009) The Sequence Alignment/Map format and SAMtools. Bioinformatics 25: 2078–2079. 19505943 10.1093/bioinformatics/btp352 PMC2723002

[23] Wang W, Carvalho B, Miller ND, Pevsner J, Chakravarti A, et al (2008) Estimating genome-wide copy number using allele-specific mixture models. Journal of computational biology a journal of computational molecular cell biology 15: 857–866. 18707534 10.1089/cmb.2007.0148 PMC2612042

[24] Morin RD, Mendez-Lago M, Mungall AJ, Goya R, Mungall KL, et al (2011) Frequent mutation of histone-modifying genes in non-hodgkin lymphoma. Nature 476: 298–303. 21796119 10.1038/nature10351 PMC3210554

[25] Li H, Ruan J, Durbin R (2008) Mapping short DNA sequencing reads and calling variants using mapping quality scores. Genome Research 18: 1851–1858. 18714091 10.1101/gr.078212.108 PMC2577856

[26] Ding J, Bashashati A, Roth A, Oloumi A, Tse K, et al (2012) Feature-based classifiers for somatic mutation detection in tumour-normal paired sequencing data. Bioinformatics 28: 167–175. 22084253 10.1093/bioinformatics/btr629 PMC3259434

