# Supplementary material for: Mutation Discovery in Regions of Segmental Cancer Genome Amplifications with CoNAn-SNV: A Mixture Model for Next Generation Sequencing of Tumors
Source: PLoS One. 2012 Aug 16;7(8):e41551. doi: 10.1371/journal.pone.0041551 (PMC3420914; doi:10.1371/journal.pone.0041551)
Supplement: Methods S1 — Validation of SNVs in lobular breast cancer. (DOC) [file pone.0041551.s001.doc]

**Methods: SeqVal01 (INX021): Validation of SNVs in lobular breast cancer**

Genomic DNA was prepared as described (Shah et al. 2009). Automated primer design was performed using Primer3 (Rozen and Skaletsky, 1998) and custom scripting. Primer pairs were designed to place the variant position within 75bps of either end of the amplicon and to be between 50-300bp in length. A total of 202 unique variant positions were targeted of which 192 yielded primer pairs that met the specified criteria. Primer pairs were independently validated by *in silco* PCR followed by BLAT against the human genome to ensure that the correct target was generated and that resulting amplicon was unique within the genome. DNA primers were synthesized in 96-well plates at a 25nmol scale with standard desalting (IDT Coralville, IA USA). Polymerase cycling reactions were set up in 96-well plates and comprised of 0.5 µM forward primer, 0.5 µM reverse primer, 1-2 ng of gDNA template, 5X Phusion HF Buffer, 0.2 µM dNTPs, 3% DMSO, and 0.4 units of Phusion DNA polymerase (NEB, Ipswich, MA, USA). Reaction plates were cycled on a MJR Peltier Thermocycler (model PTC-225) with cycling conditions of a denaturation step at 98 °C for 30 sec, followed by 35 cycles of [98°C for 10 sec, 69°C for 15 sec, 72°C for 15 sec] and a final extension step at 72°C for 10 min. PCR reactions were visualized on 3% agarose (NuSieve) gels for 2hrs at 170V to assess PCR success. Successful reactions were manually pooled (4ul per well) by template and subjected to Illumina library construction using a modified paired-end protocol (Illumina, Hayward, USA). This involved A-tailing of the amplicons and ligation to Illumina PE adapters. Adapter-ligated products were purified on Qiaquick spin columns (Qiagen, Valencia, CA, USA) and PCR-amplified using Phusion DNA polymerase (NEB, Ipswich, MA, USA) in 10 cycles using PE primer 1.0 (Illumina) and a custom multiplexing PCR Primer [5'-CAAGCAGAAGACGGCATACGAGATNNNNNNCGGTCTCGGCATTCCTGC TGAACCGCTCTTCCGATCT-3’] where “NNNNNN” was replaced with the following unique fault tolerant hexamer barcodes: pleural effusion metastatic tumor, “CGTGAT”; buffy coat, “ACATCG”; and primary tumor “GCCTAA”. PCR products of the desired size range were purified away from adapter ligation artifacts using 8% PAGE gels, pooled and DNA quality was assessed and quantified using an Agilent DNA 1000 series II assay (Agilent, Santa Clara CA, USA) and Nanodrop 7500 spectrophotometer (Nanodrop, Wilmington, DE, USA) and subsequently diluted to 10nM. The final concentration was confirmed using a Quant-iT dsDNA HS assay kit and Qubit fluorometer (Invitrogen, Carlsbad, CA, USA). For sequencing, clusters were generated on the Illumina cluster station using v4 cluster reagents and paired-end 75bp reads generated using v4 sequencing reagents on the Illumina GA_ii_ platform following the manufacturer’s instructions. Between the paired 75bp reads a third 7 base pair read was performed using the following custom sequencing primer [5- GATCGGAAGAGCGGTTCAGCAGGAATGCCGAGACCG] to sequence the hexamer barcode. Image analysis, base-calling and error calibration was performed using v1.60 of Illumina’s Genome analysis pipeline.

Refs

Steve Rozen, Helen J. Skaletsky (1998) Primer3. Code available at http://www-genome.wi.mit.edu/genome_software/other/primer3.html.
