# Supplementary material for: Mutation Discovery in Regions of Segmental Cancer Genome Amplifications with CoNAn-SNV: A Mixture Model for Next Generation Sequencing of Tumors
Source: PLoS One. 2012 Aug 16;7(8):e41551. doi: 10.1371/journal.pone.0041551 (PMC3420914; doi:10.1371/journal.pone.0041551)
Supplement: Figure S1 — Copy number annotations for all chromosome made by OncoSNP. (PDF) [file pone.0041551.s002.pdf]

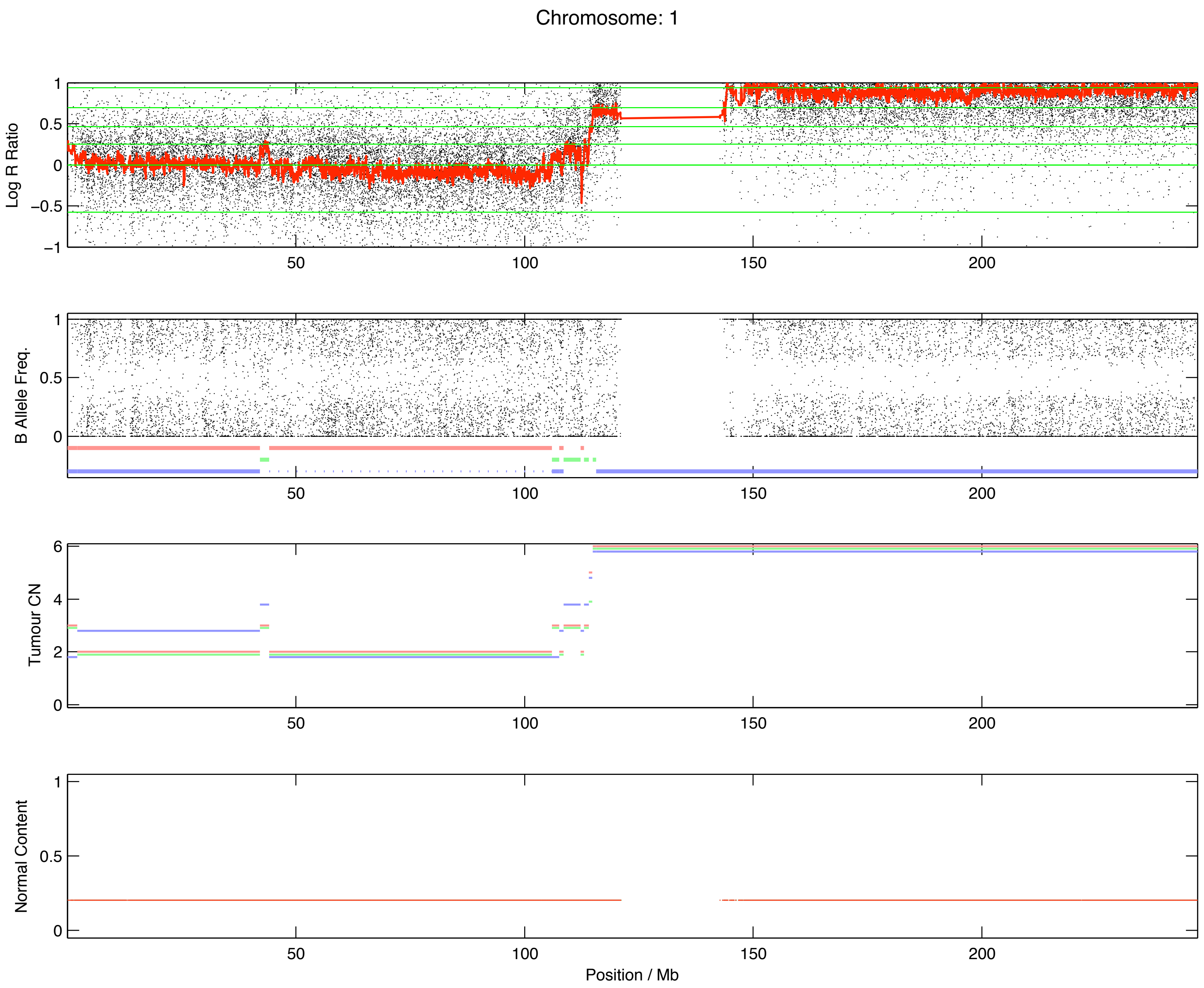

Chromosome: 1
Log R Ratio
B Allele Freq.
Tumour CN
Normal Content
Position / Mb
1
0.5
0
−0.5
−1
6
4
2
50
100
150
200

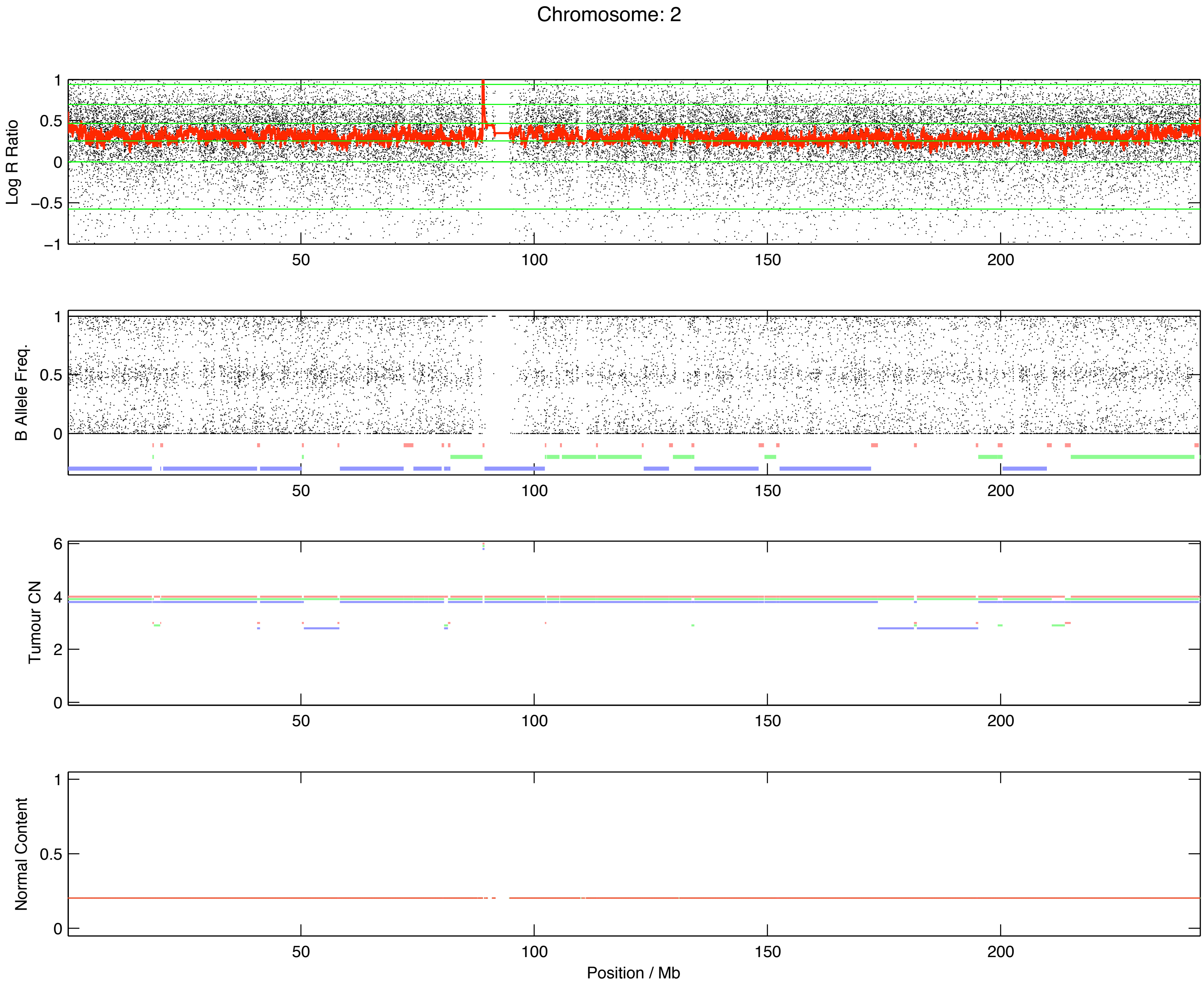

Chromosome: 2
Log R Ratio
B Allele Freq.
Tumour CN
Normal Content
Position / Mb

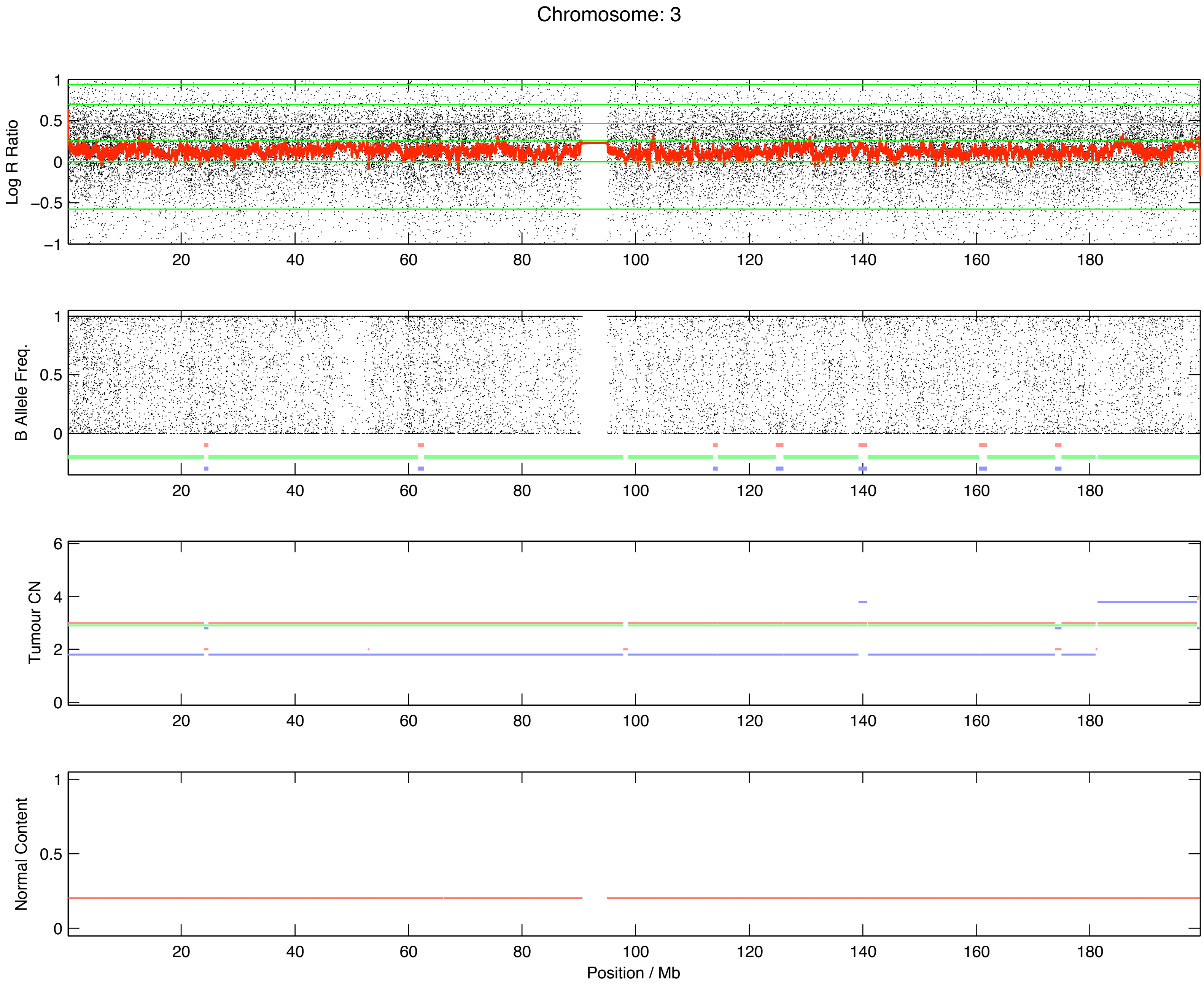

Chromosome: 3
Log R Ratio
B Allele Freq.
Tumour CN
Normal Content
Position / Mb

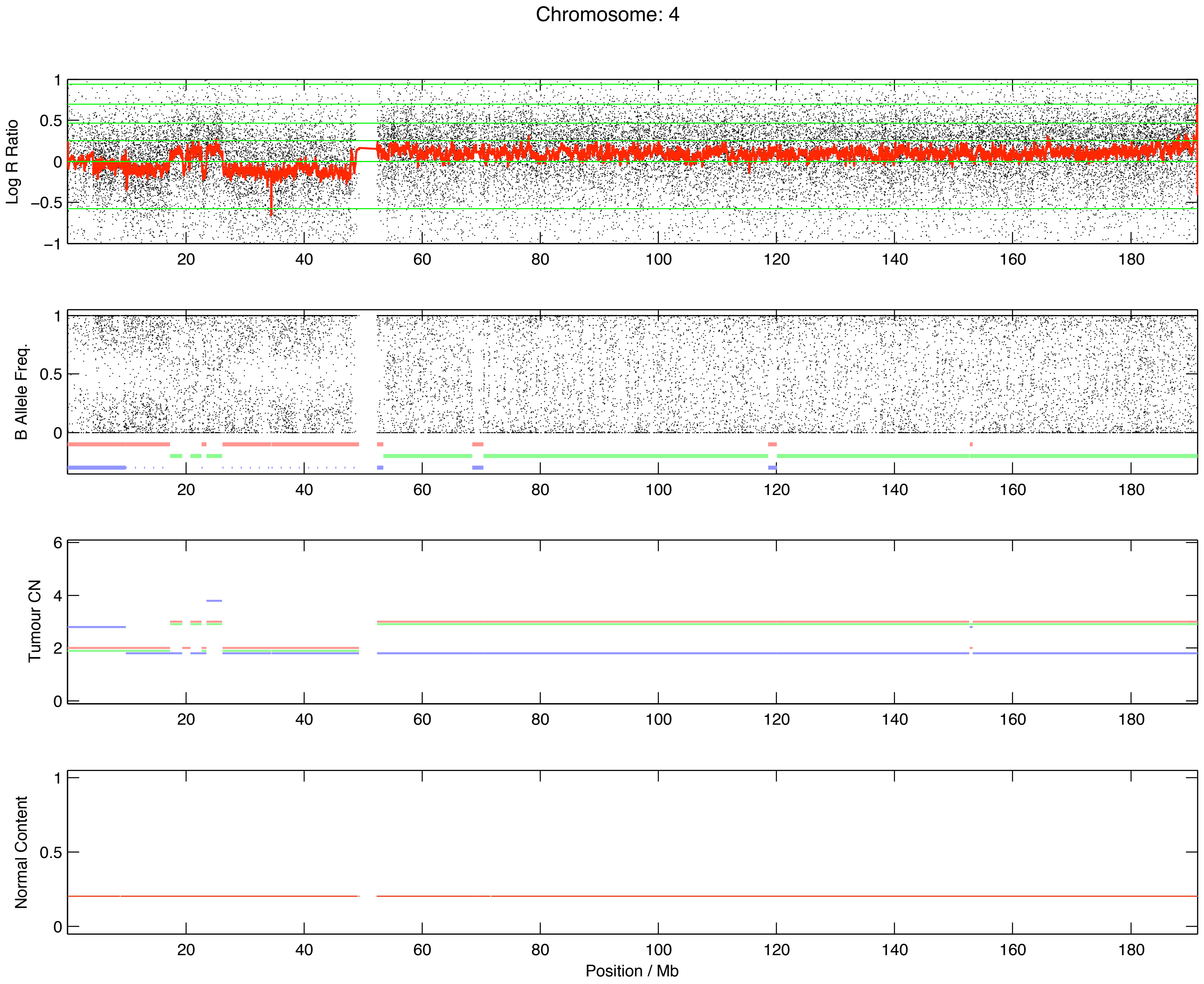

Chromosome: 4
Log R Ratio
B Allele Freq.
Tumour CN
Normal Content
Position / Mb

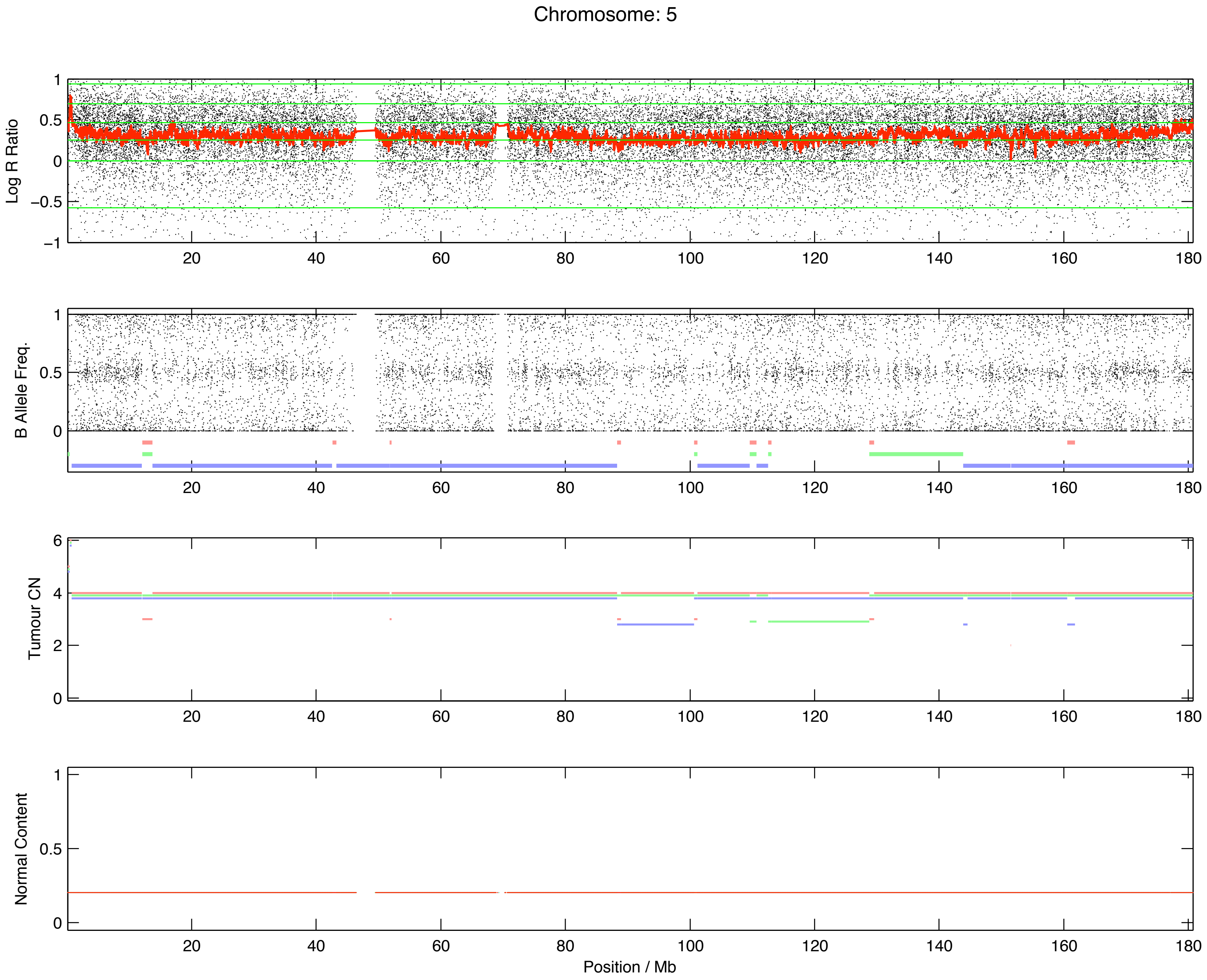
Chromosome: 5
Log R Ratio
1
0.5
0
−0.5
−1
20
40
60
80
100
120
140
160
180
B Allele Freq.
Tumour CN
6
4
2
Normal Content
Position / Mb

Chromosome: 6

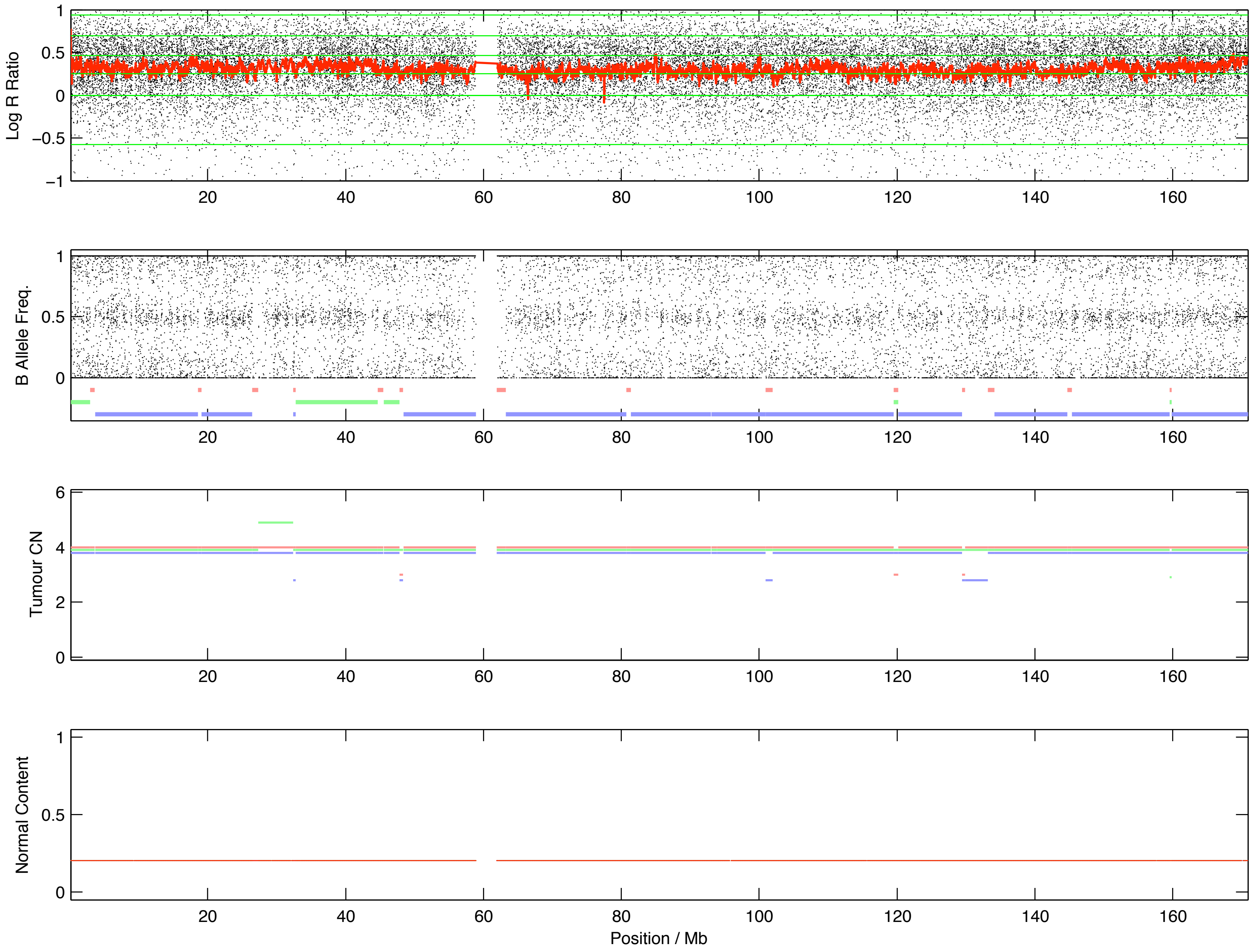

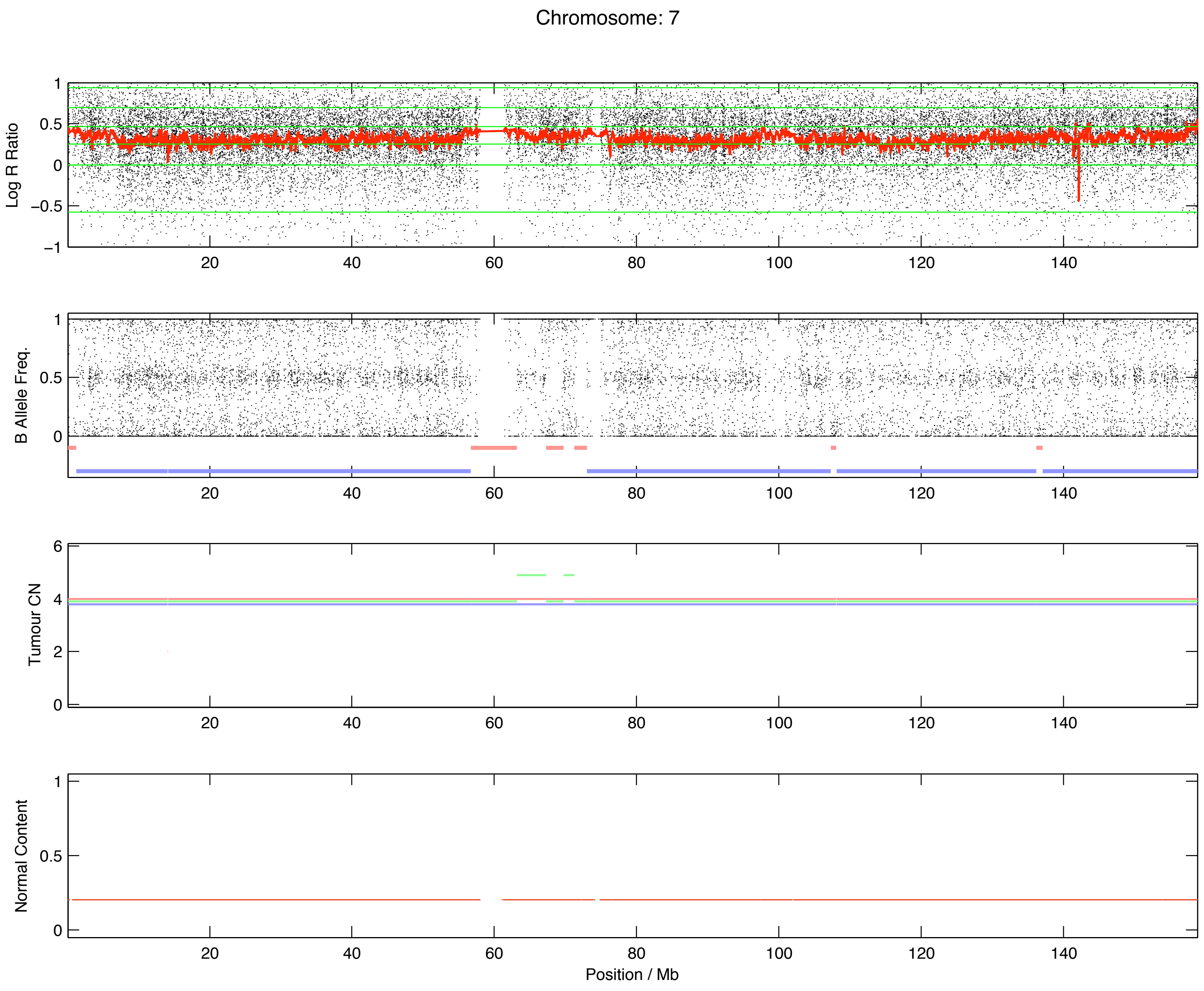
Chromosome: 7
Log R Ratio
B Allele Freq.
Tumour CN
Normal Content
Position / Mb
1
0.5
0
−0.5
−1
6
4
2
20
40
60
80
100
120
140

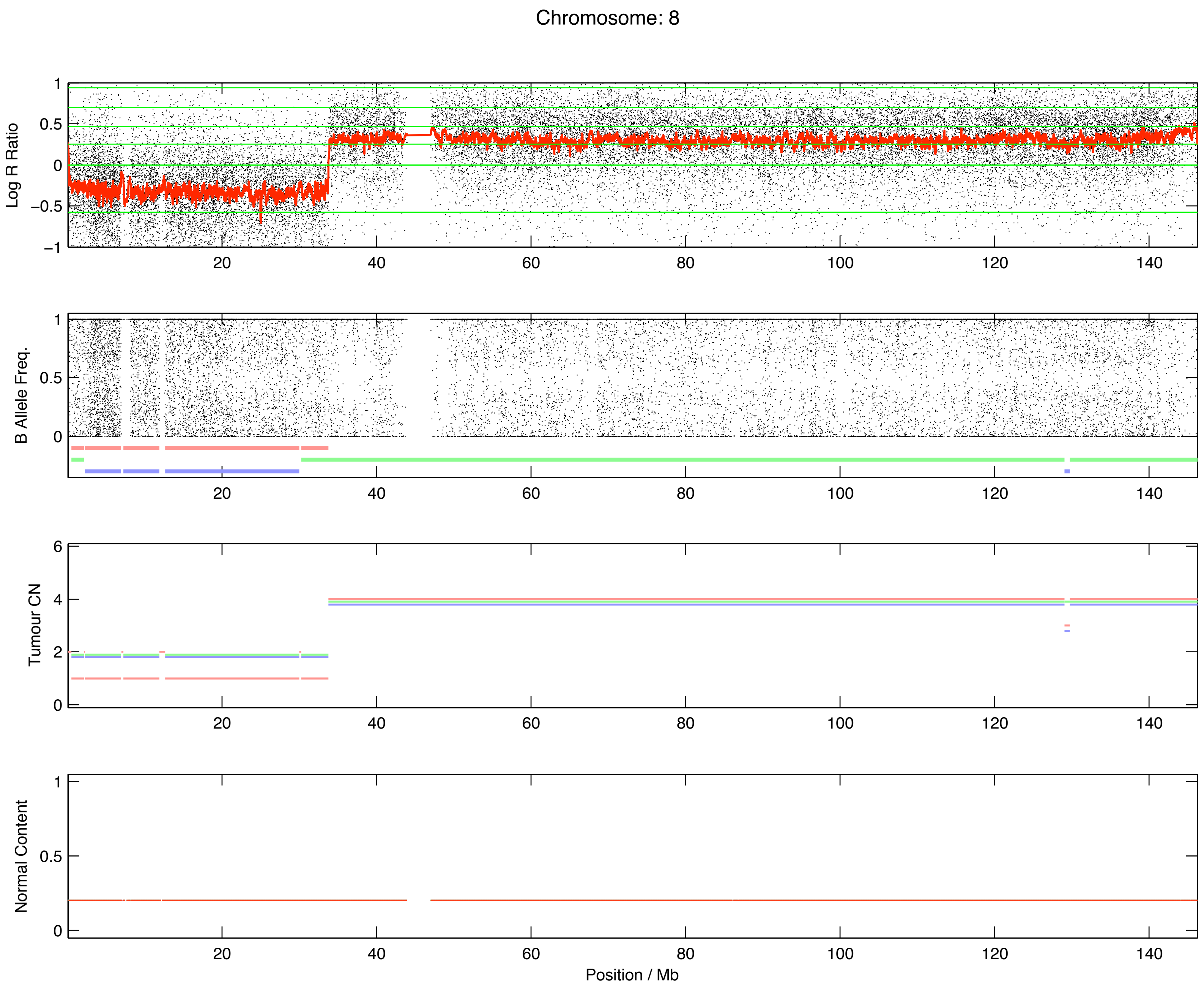

Chromosome: 8
Log R Ratio
B Allele Freq.
Tumour CN
Normal Content
Position / Mb

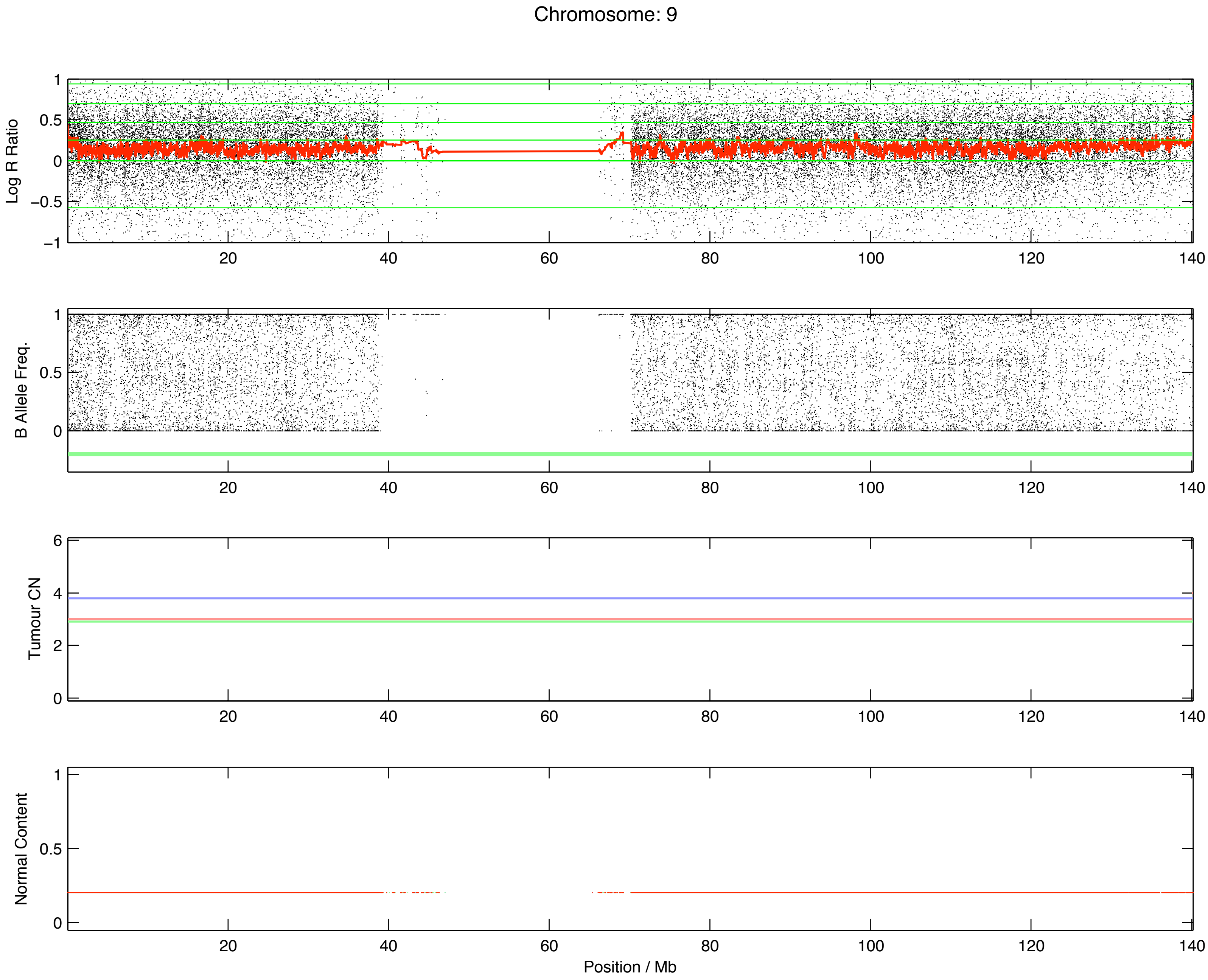

Chromosome: 9
Log R Ratio
1
0.5
0
−0.5
−1
B Allele Freq.
Tumour CN
6
4
2
Normal Content
20
40
60
80
100
120
140
Position / Mb

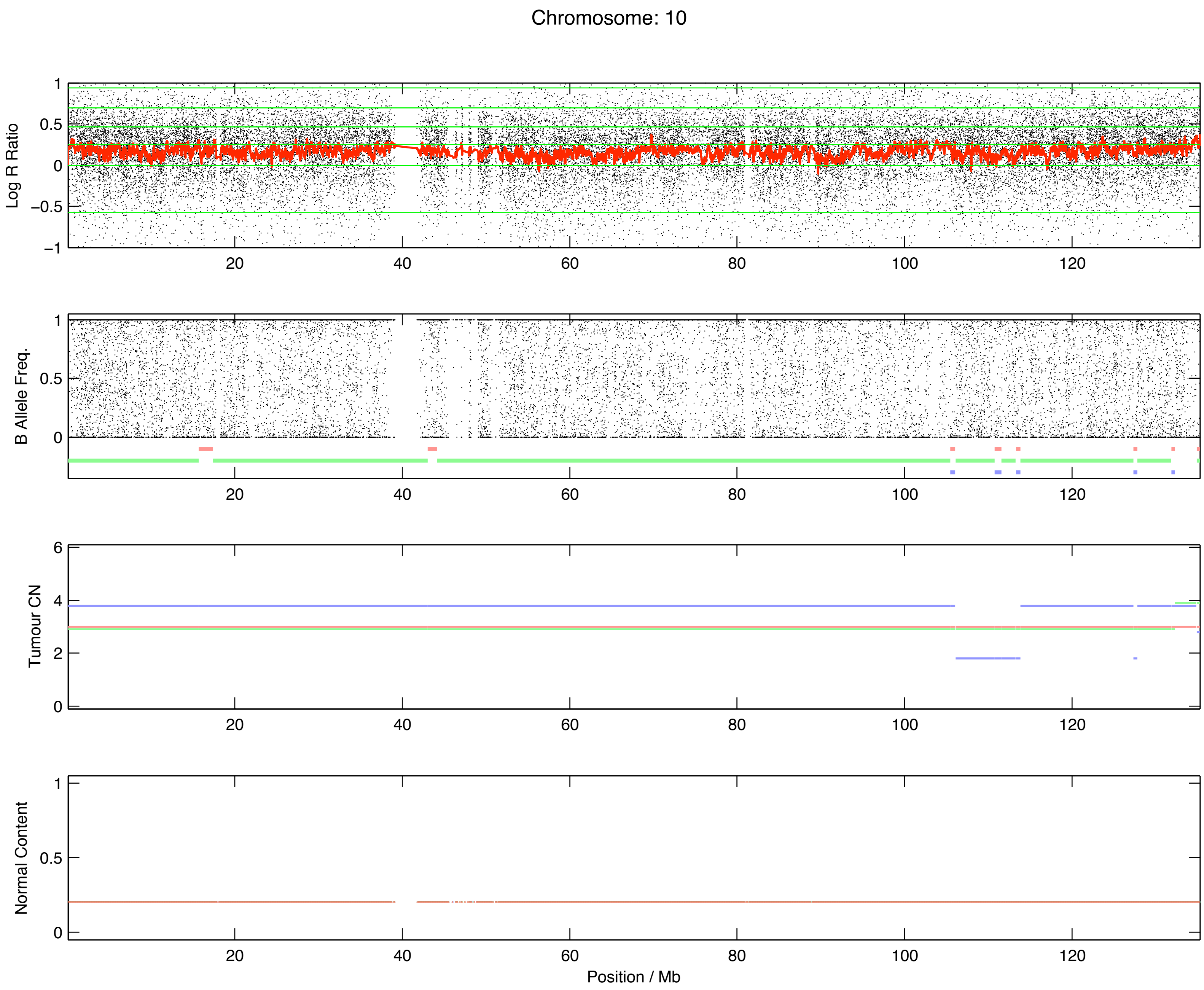
Chromosome: 10
Log R Ratio
B Allele Freq.
Tumour CN
Normal Content
Position / Mb

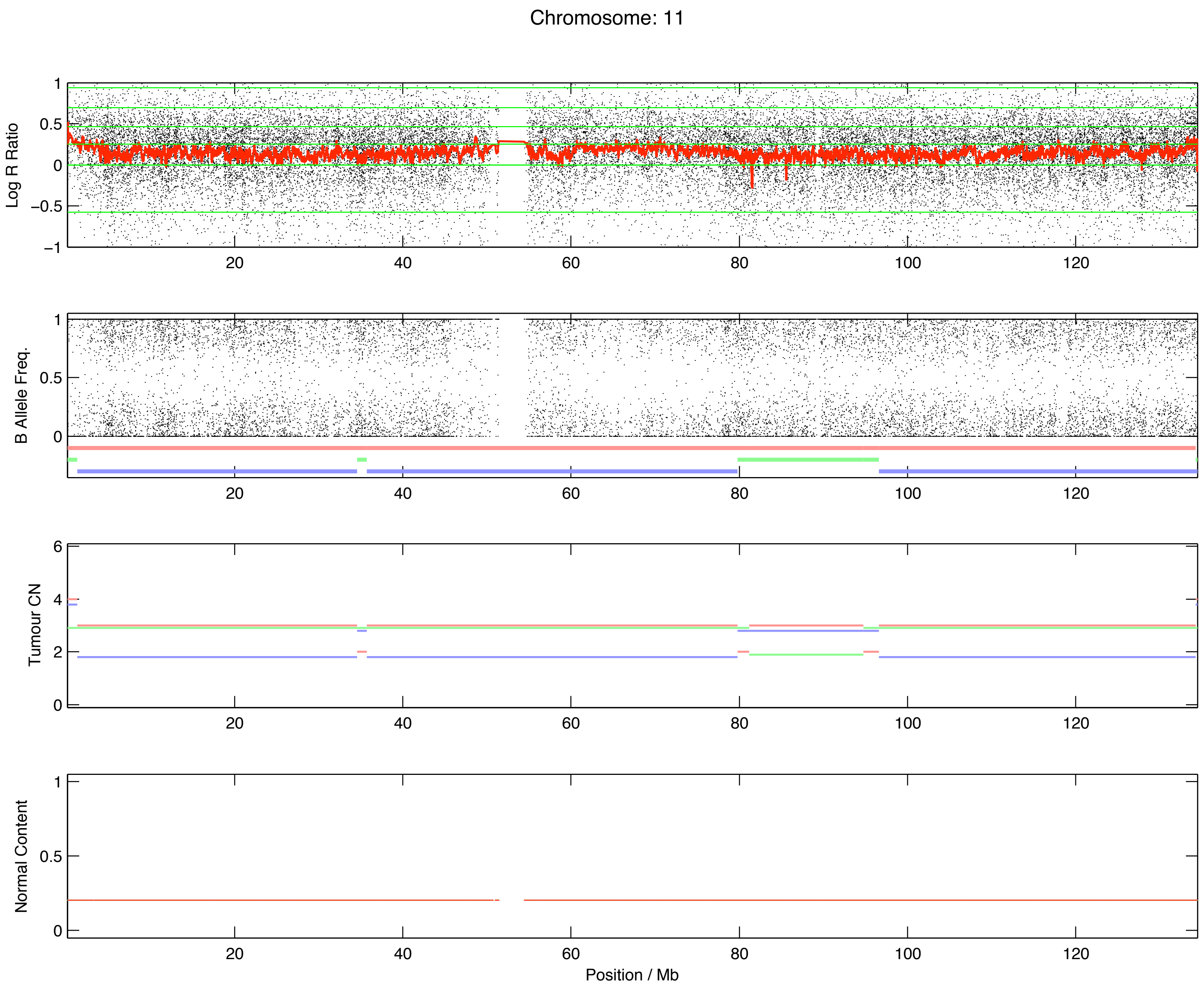

Chromosome: 11
Log R Ratio
B Allele Freq.
Tumour CN
Normal Content
Position / Mb
1
0.5
0
−0.5
−1
6
4
2
20
40
60
80
100
120

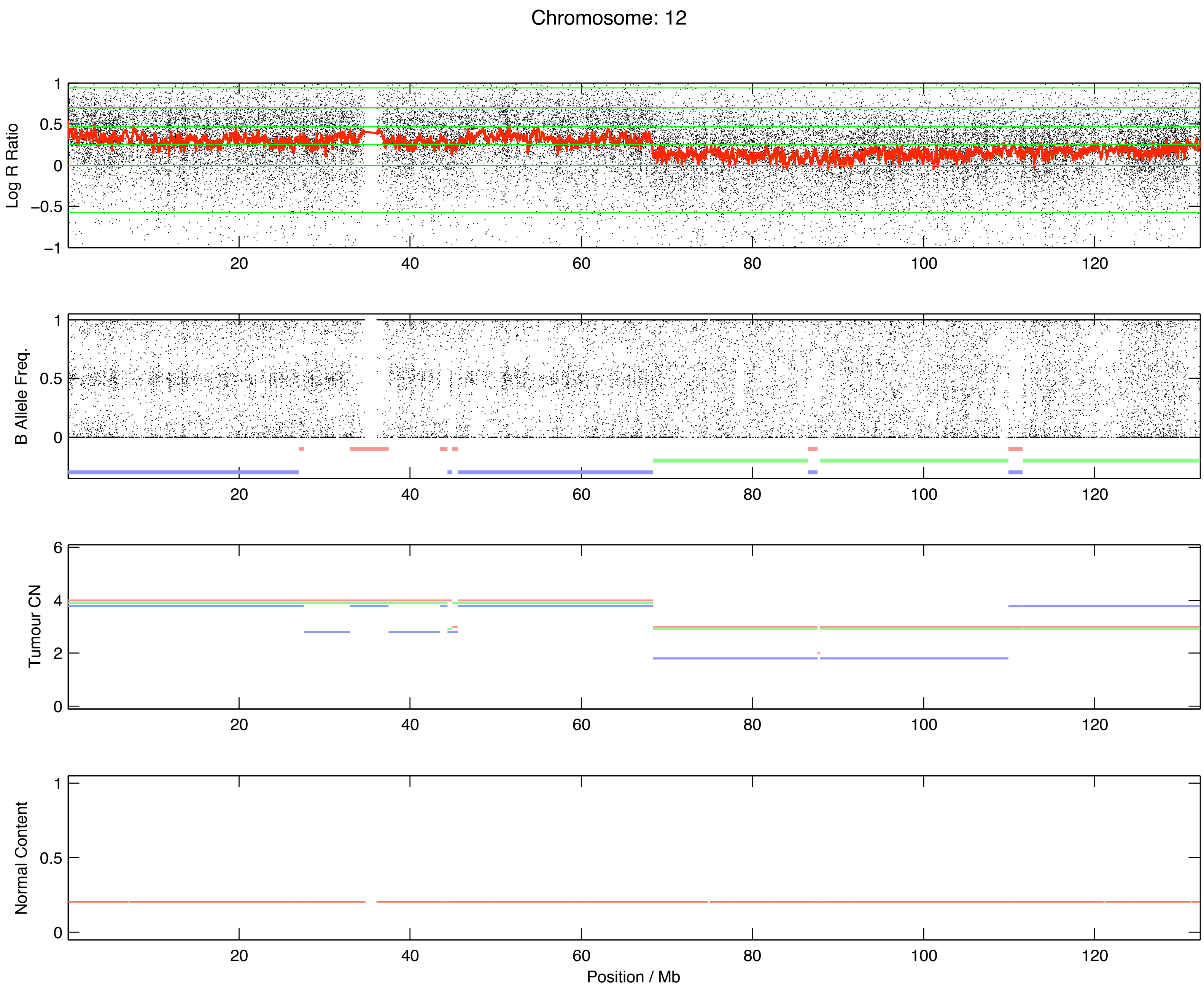

Chromosome: 12
Log R Ratio
B Allele Freq.
Tumour CN
Normal Content
Position / Mb

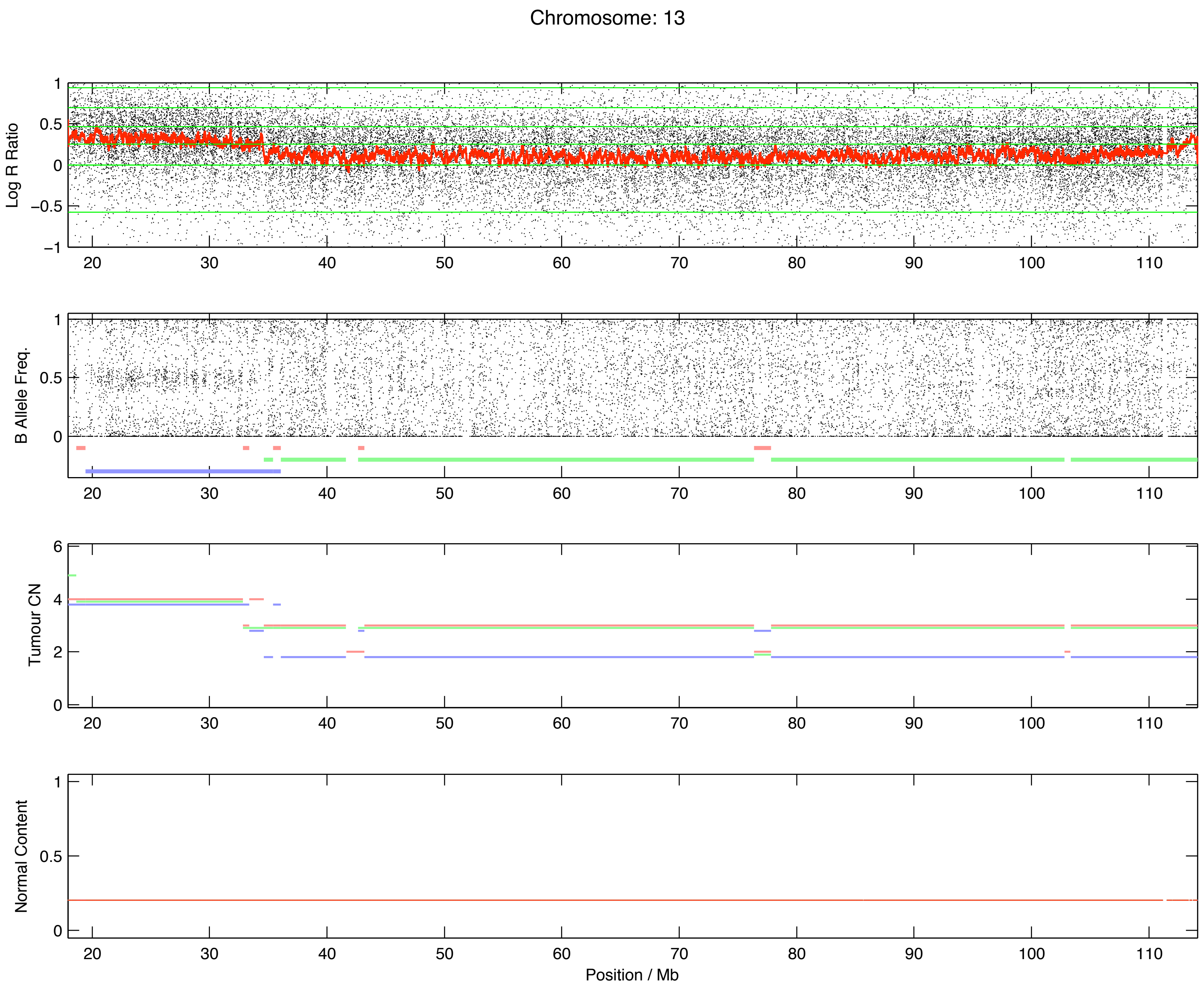

Chromosome: 13
Log R Ratio
B Allele Freq.
Tumour CN
Normal Content
Position / Mb

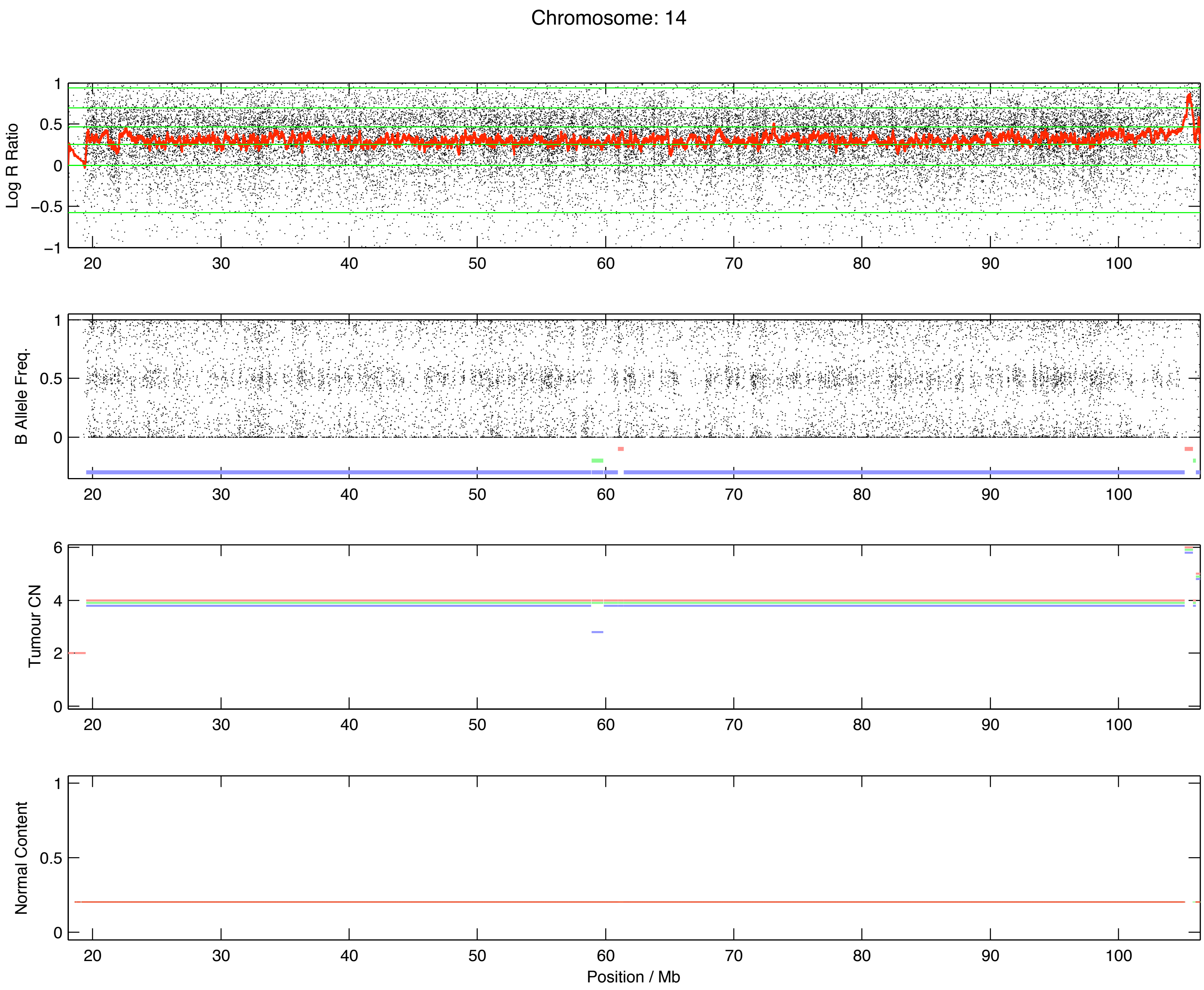
Chromosome: 14
Log R Ratio
B Allele Freq.
Tumour CN
Normal Content
Position / Mb
1
0.5
0
−0.5
−1
6
4
2
20
30
40
50
60
70
80
90
100

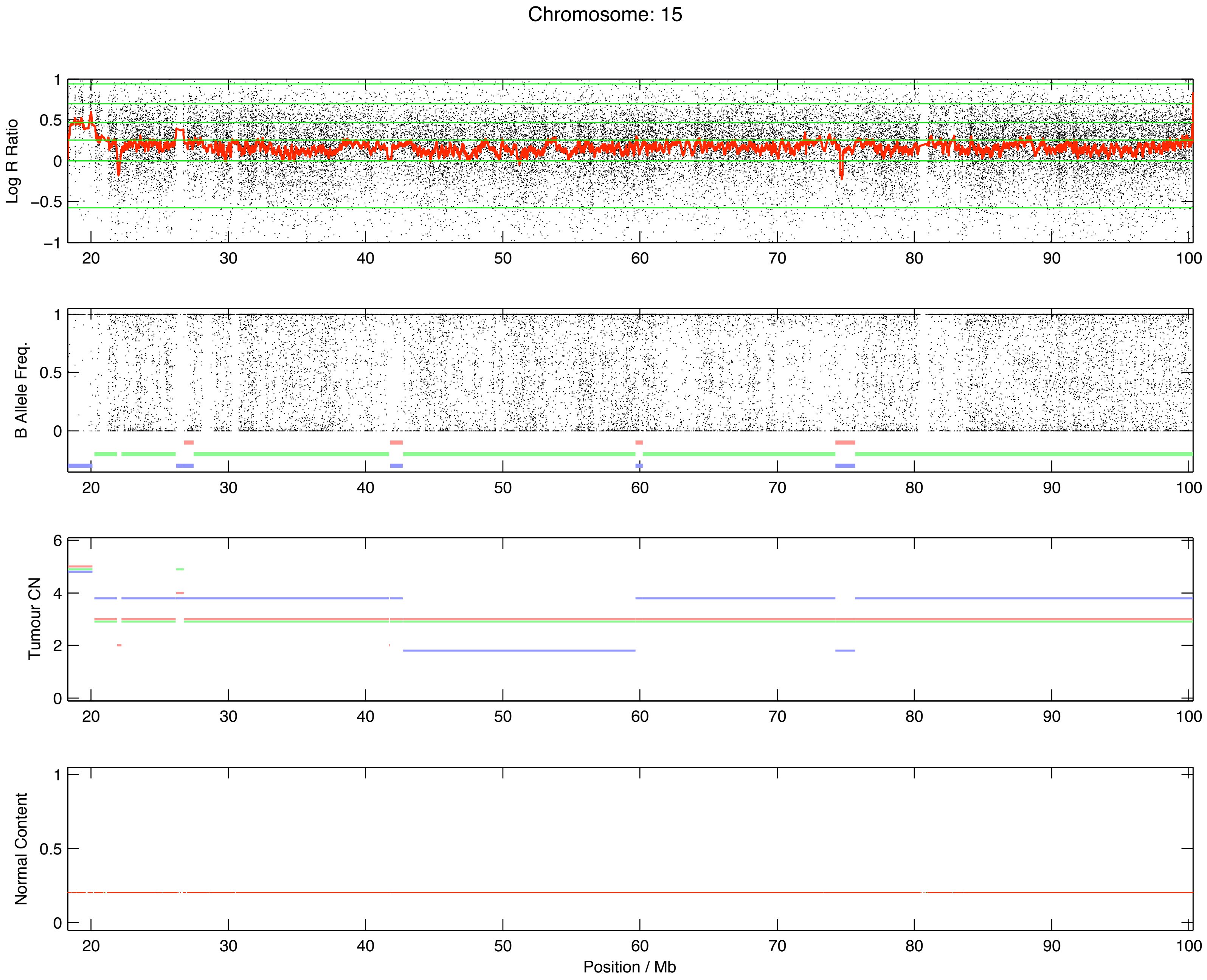

Chromosome: 15
Log R Ratio
1
0.5
0
−0.5
−1
B Allele Freq.
Tumour CN
6
4
2
Normal Content
20
30
40
50
60
70
80
90
100
Position / Mb

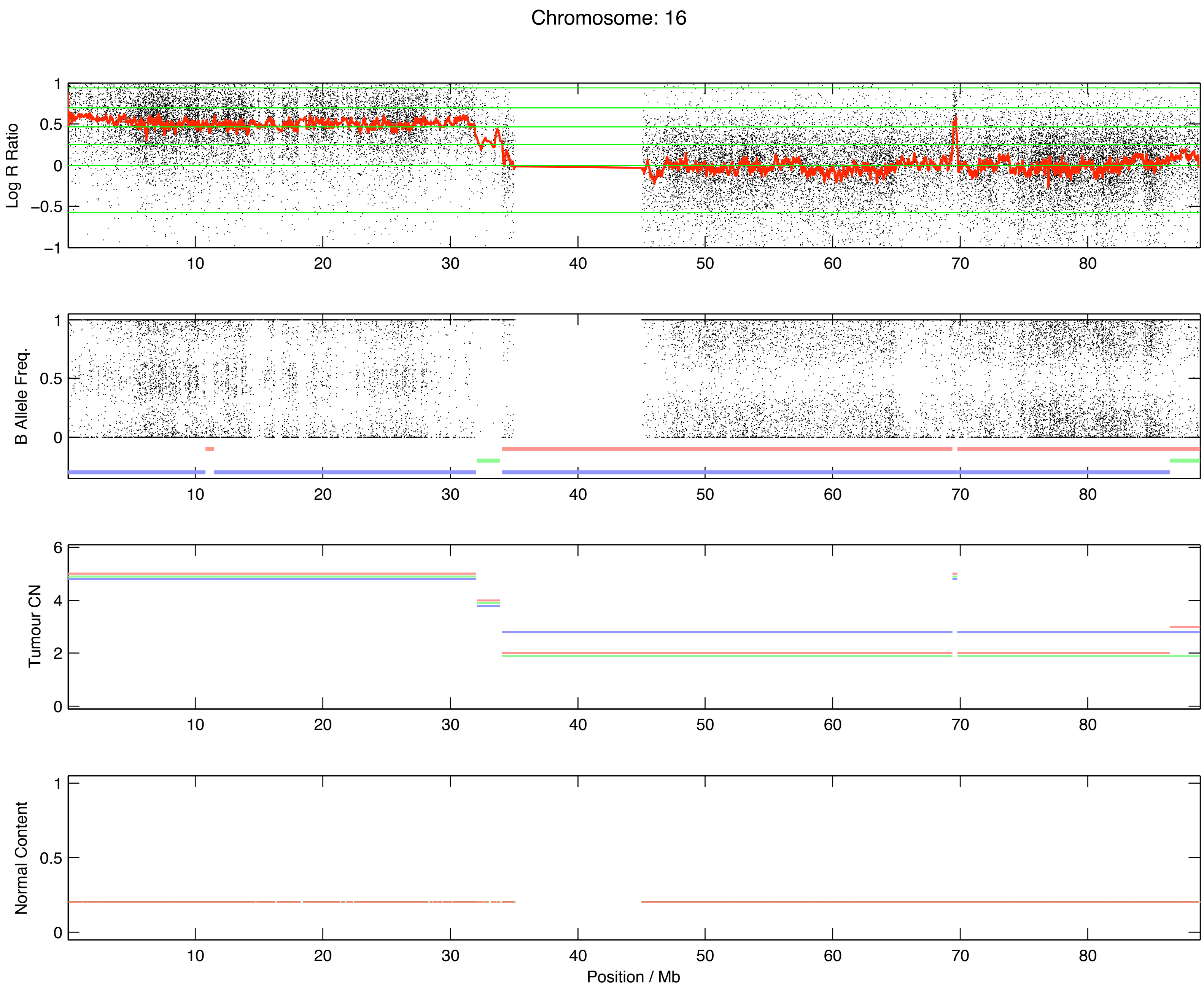

Chromosome: 16
Log R Ratio
B Allele Freq.
Tumour CN
Normal Content
Position / Mb

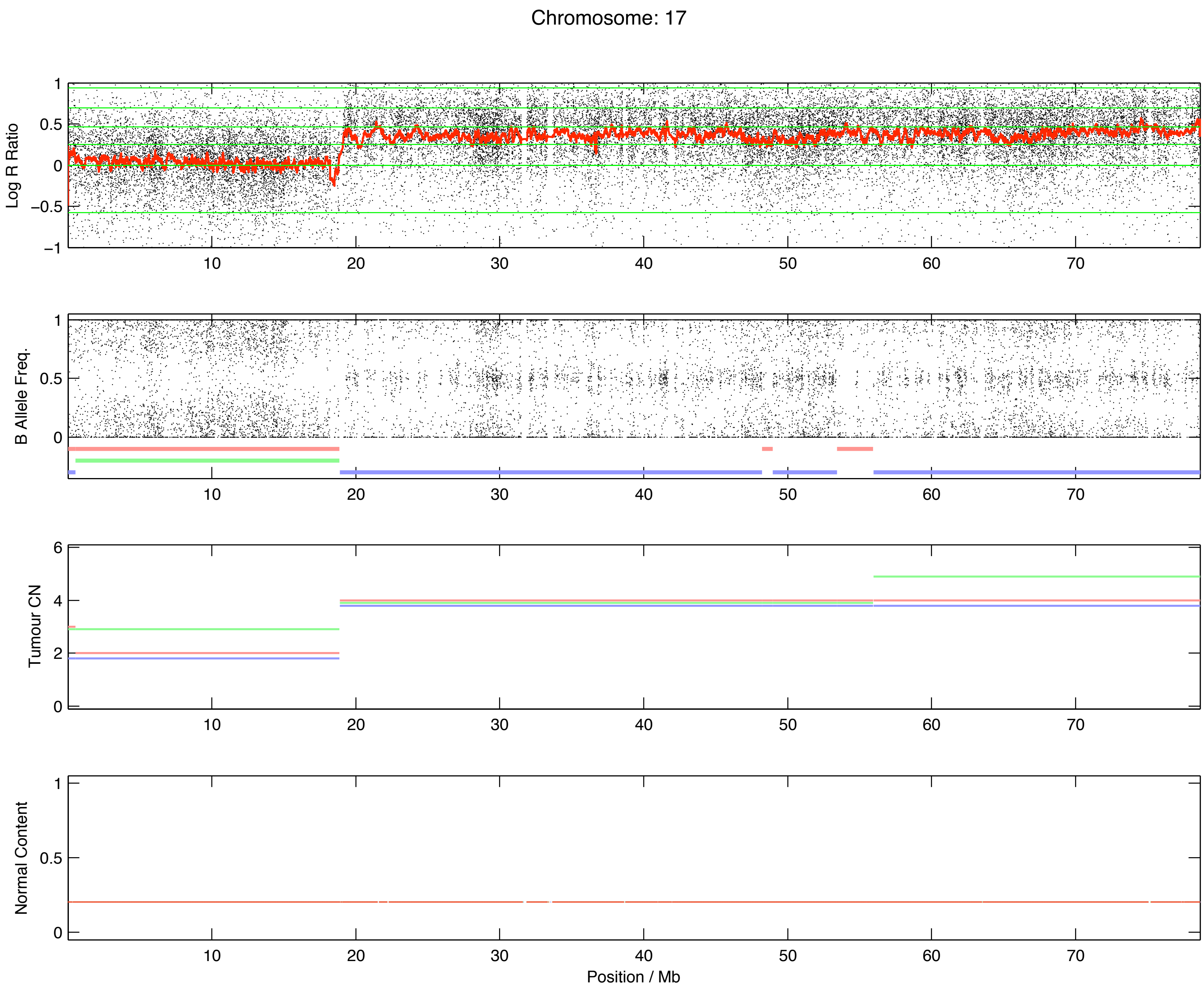
Chromosome: 17
Log R Ratio
1
0.5
0
−0.5
−1
10
20
30
40
50
60
70
B Allele Freq.
1
0.5
0
Tumour CN
6
4
2
0
Normal Content
1
0.5
0
Position / Mb

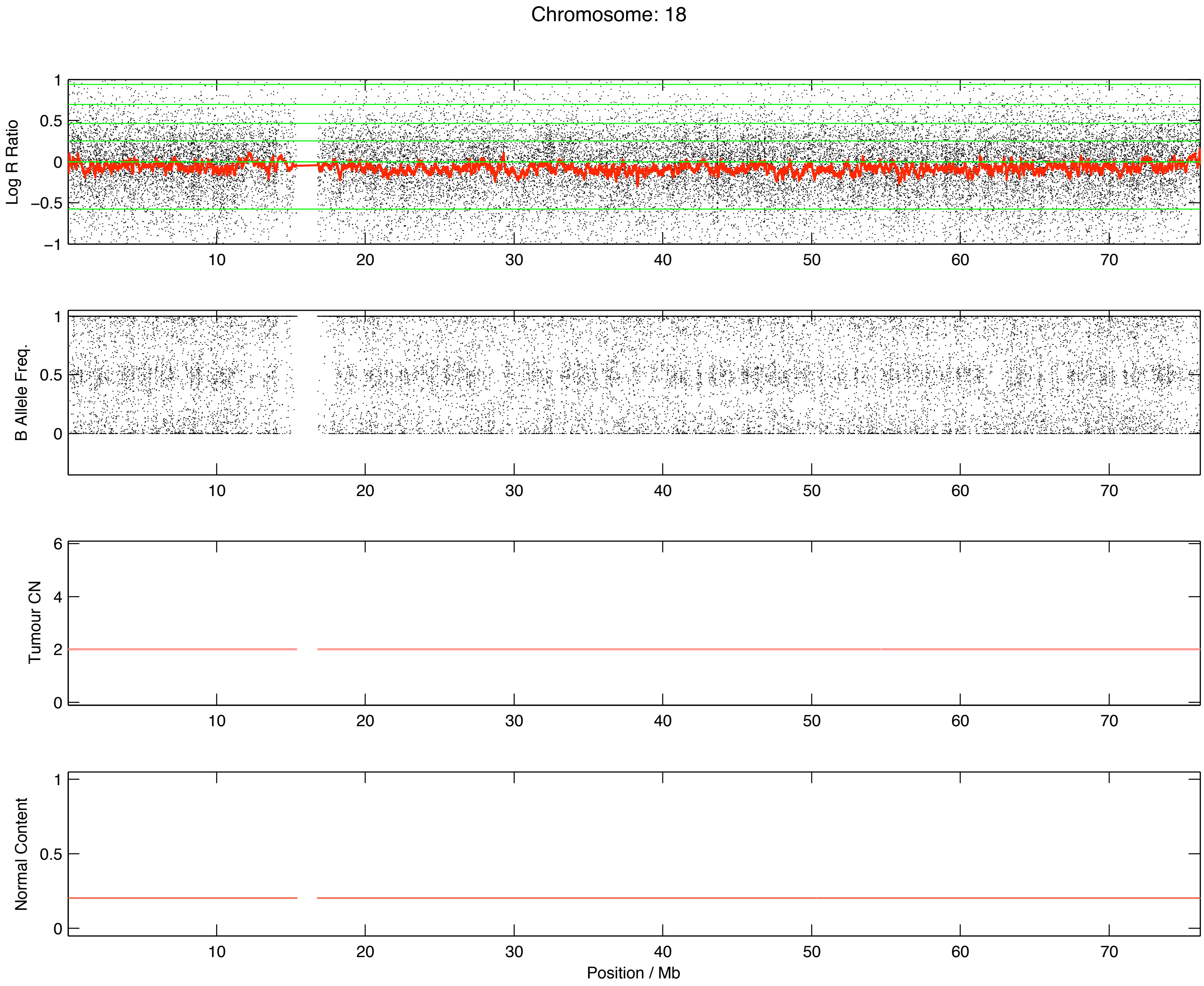

Chromosome: 18
Log R Ratio
B Allele Freq.
Tumour CN
Normal Content
Position / Mb

Chromosome: 19

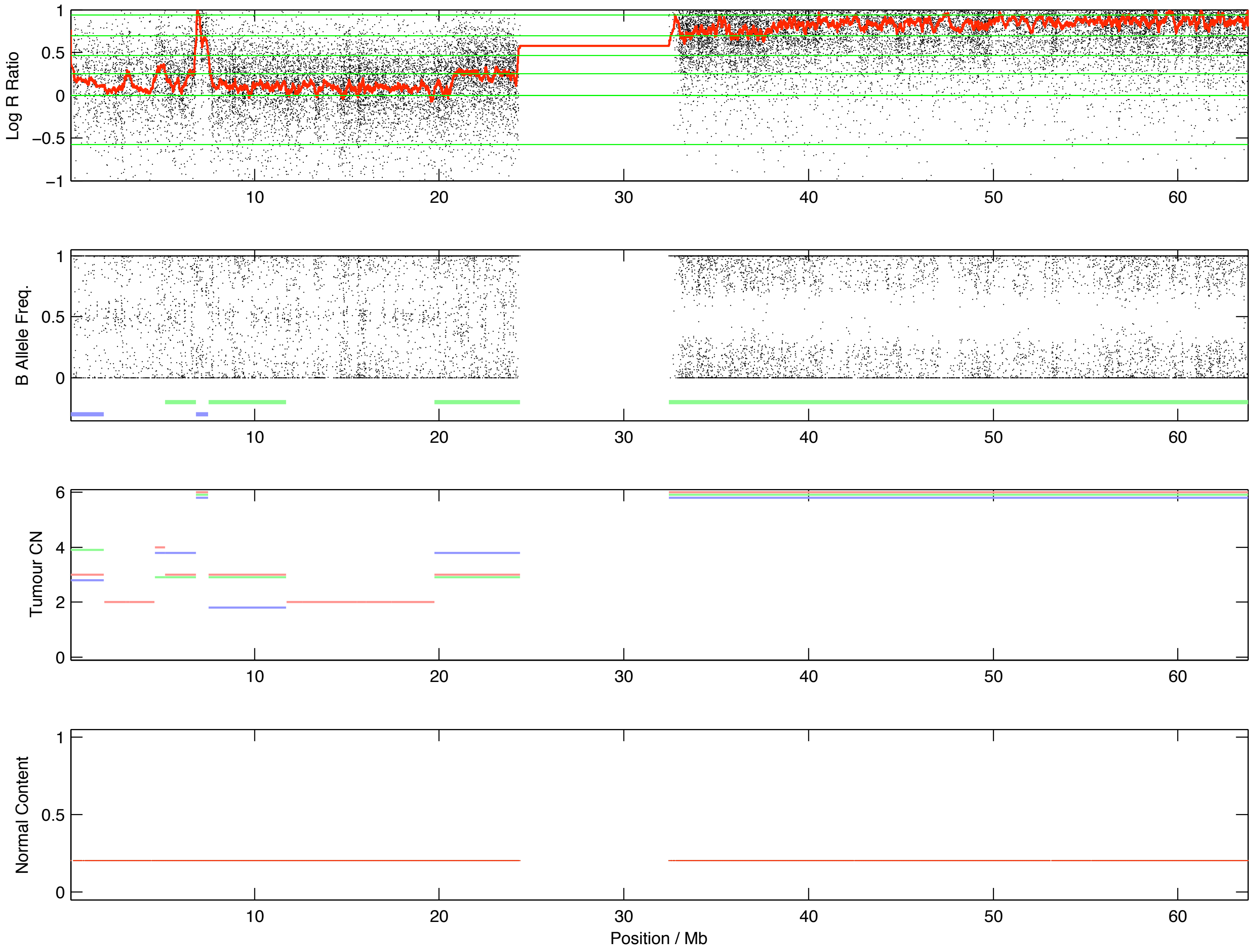

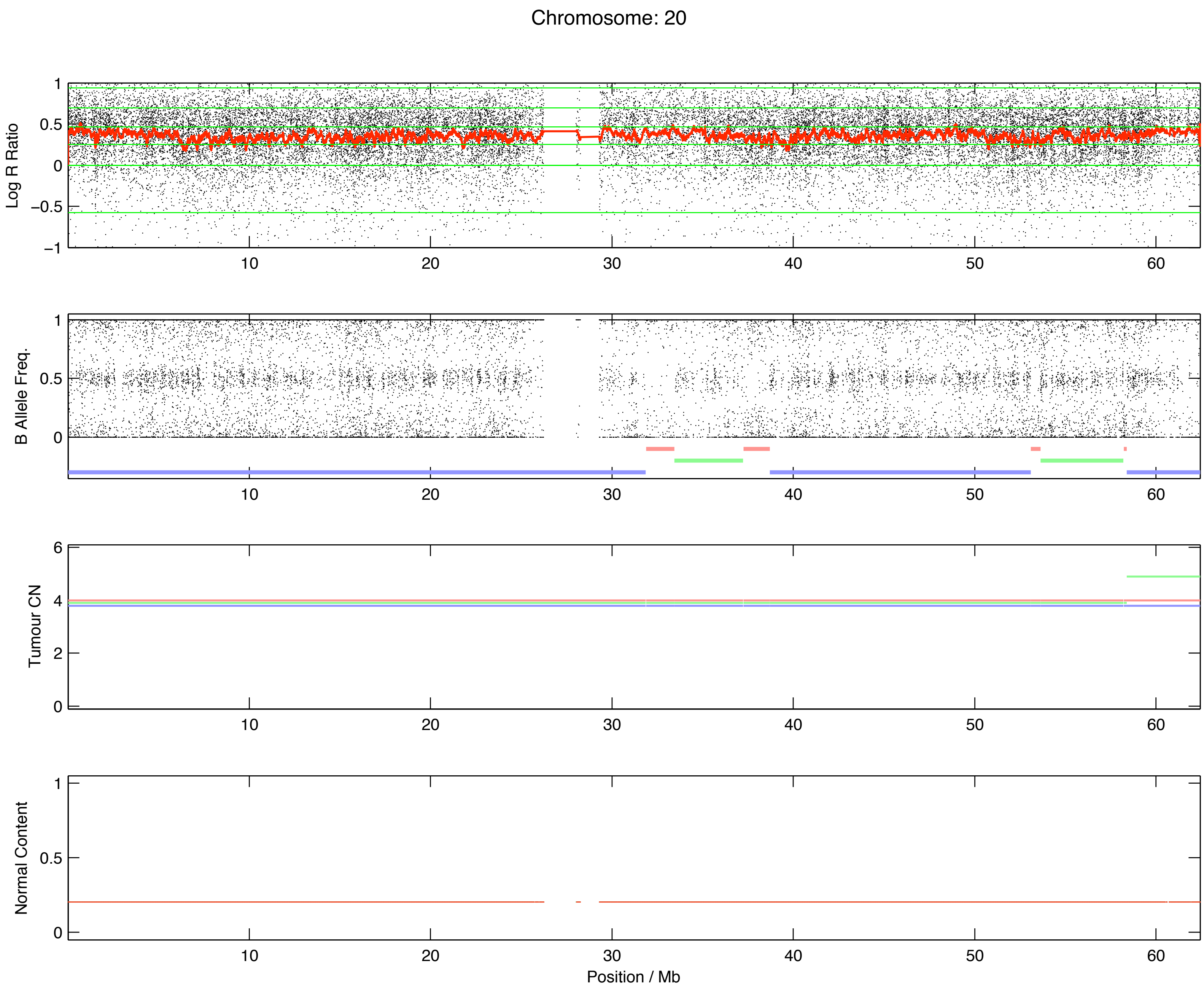

Chromosome: 20
Log R Ratio
B Allele Freq.
Tumour CN
Normal Content
Position / Mb

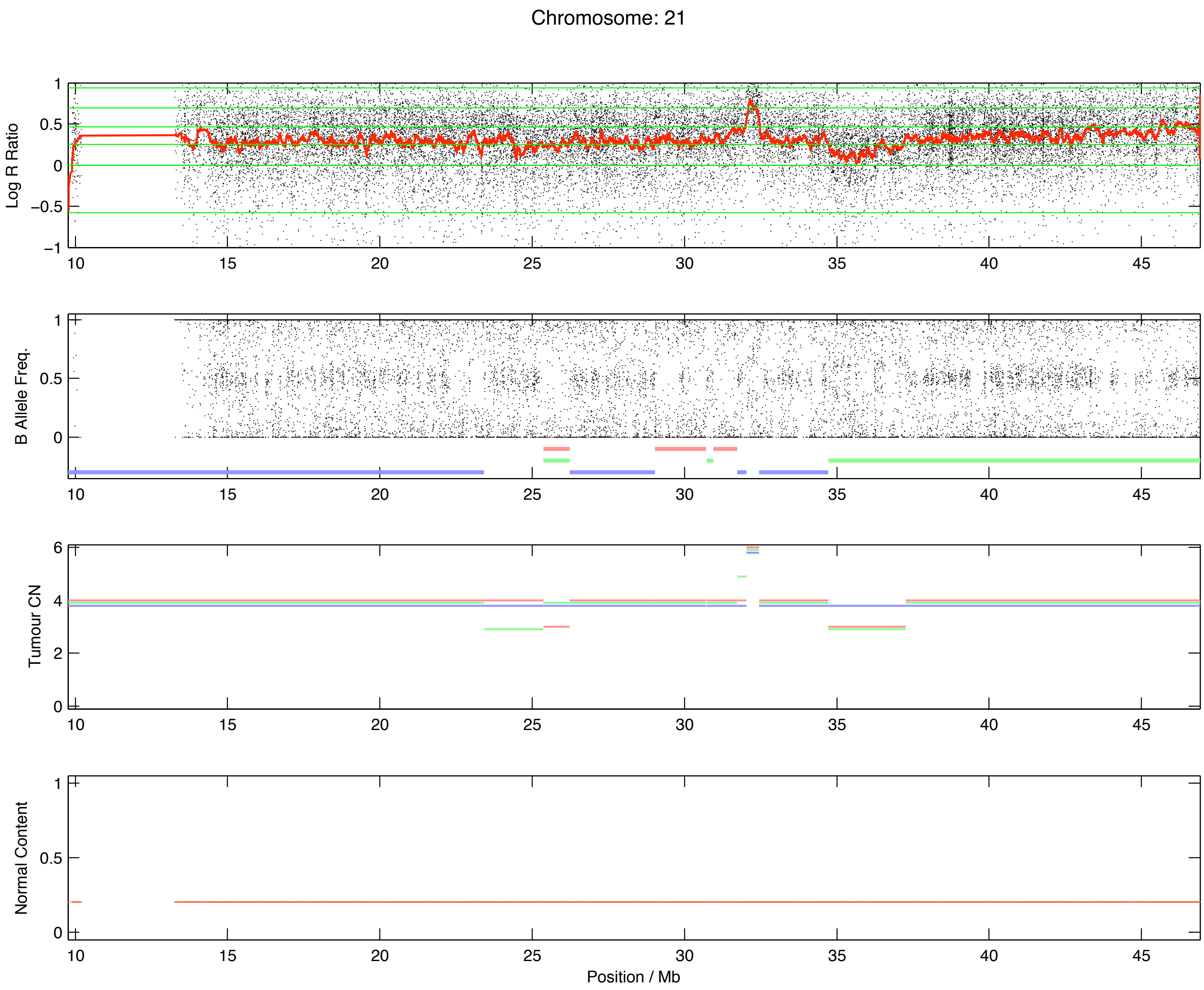
Chromosome: 21
Log R Ratio
B Allele Freq.
Tumour CN
Normal Content
Position / Mb
10
15
20
25
30
35
40
45

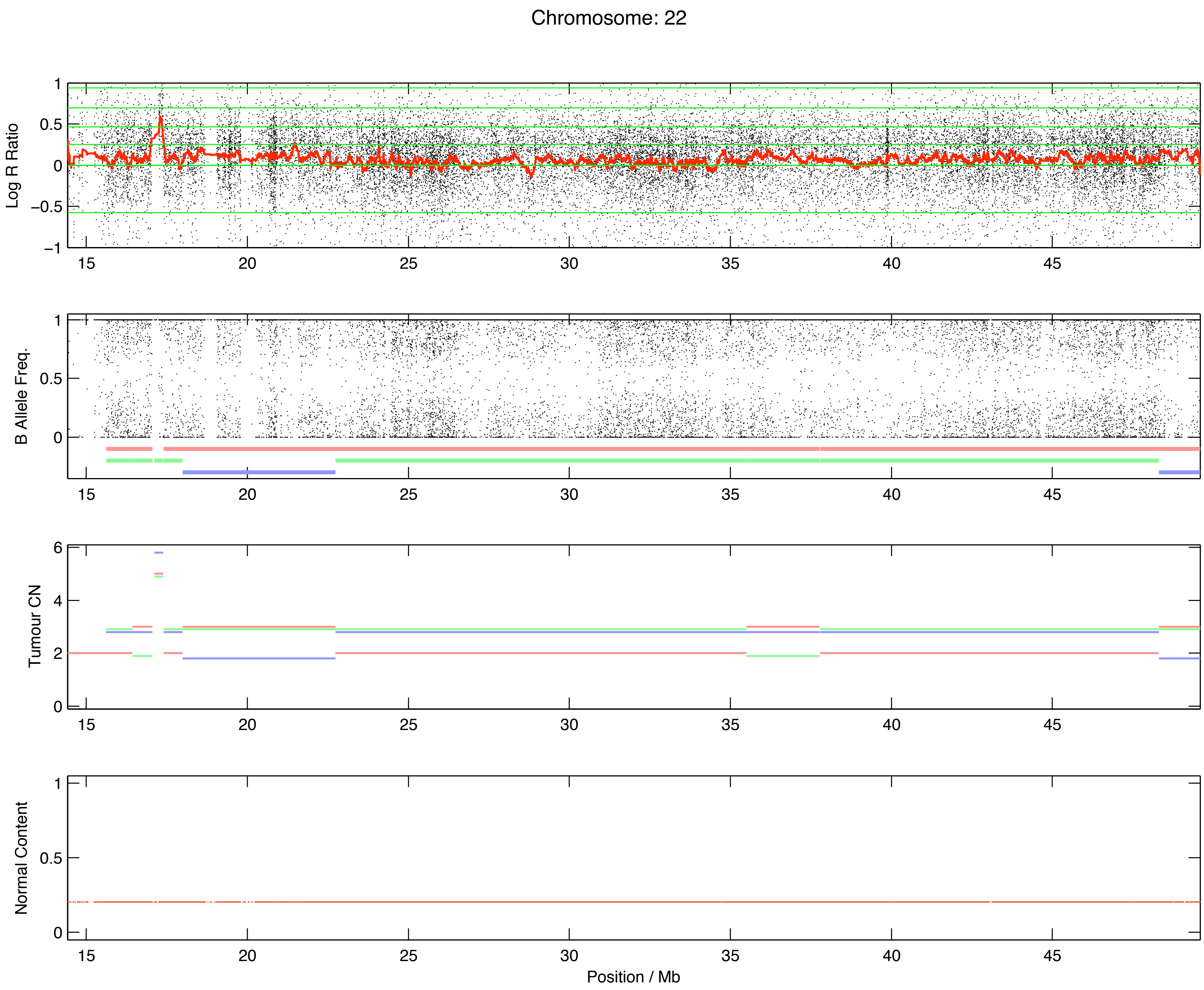

Chromosome: 22
Log R Ratio
B Allele Freq.
Tumour CN
Normal Content
Position / Mb
