# Supplementary figures and images for: Mutation Discovery in Regions of Segmental Cancer Genome Amplifications with CoNAn-SNV: A Mixture Model for Next Generation Sequencing of Tumors
Source: PLoS One. 2012 Aug 16;7(8):e41551. doi: 10.1371/journal.pone.0041551 (PMC3420914; doi:10.1371/journal.pone.0041551)

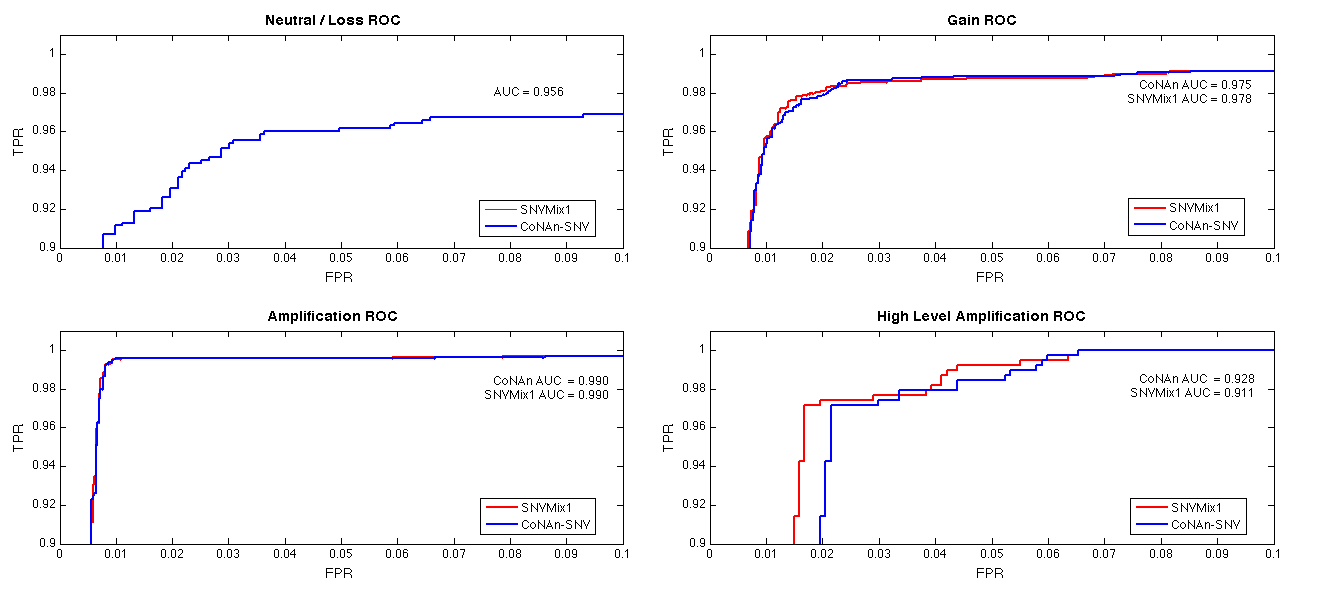

Supplement: Figure S2 — ROC for performance evaluation using CRLMM broken down by CNA state. (TIF) [file pone.0041551.s003.tif]

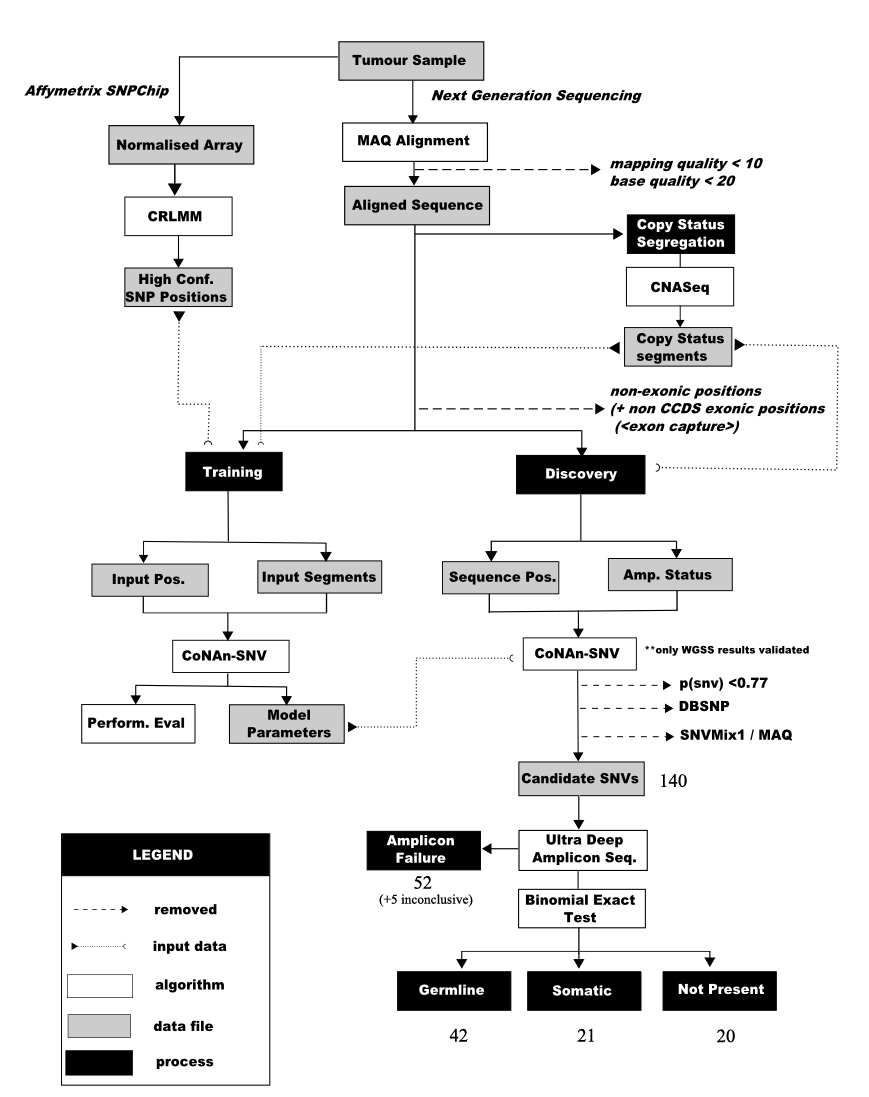

Supplement: Figure S3 — Full variant discovery pipeline. (TIF) [file pone.0041551.s004.tif]

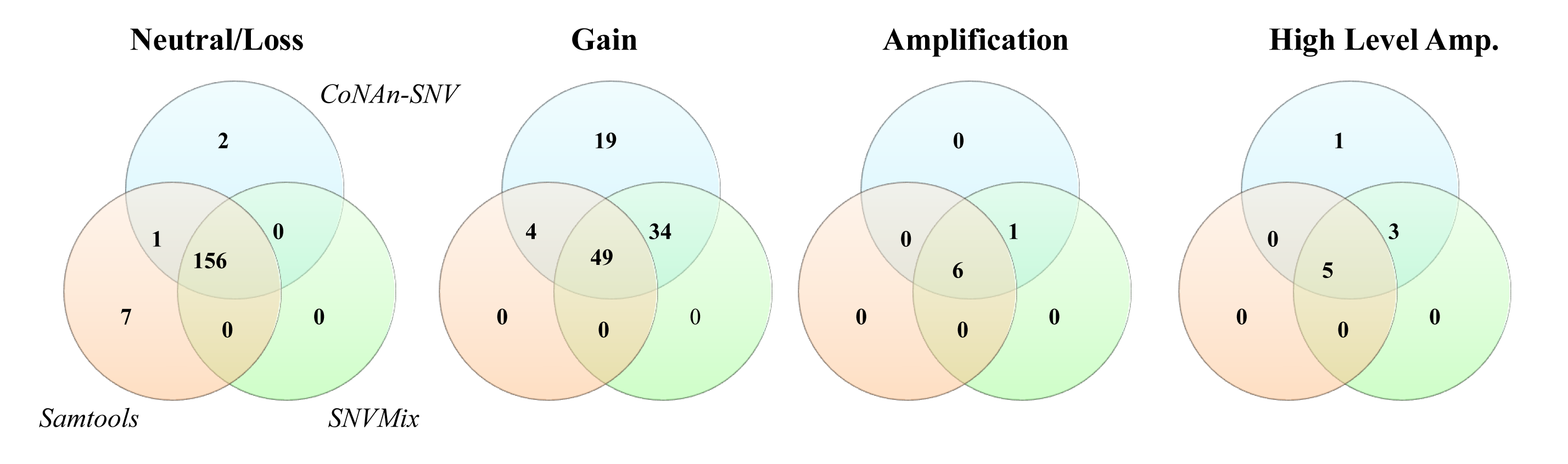

Supplement: Figure S4 — MutationSeq somatic variant results for lymphoma. Predicted variants with a probability of 0.5 or greater for being a somatic variants (probability assigned by MutationSeq) are shown in a lymphoma tumor for CoNAn-SNV, SNVMix, and the samtools variants caller. There is a high degree of concordance between the three methods, however CoNAn-SNV finds the most unique variants, especially in Gain states. (TIF) [file pone.0041551.s005.tif]
